# In vitro and in vivo evaluation of oleuropein loaded hyalurosomes for diabetic foot ulcer healing

**DOI:** 10.1038/s41598-026-42804-5

**Published:** 2026-03-26

**Authors:** Ahmed I. Elgendy, Ahmed O. El-Gendy, Heba M. Aboud, Fatma I. Abo El-Ela, Mohamed I. Zanaty

**Affiliations:** 1 Biotechnology and Life Sciences Department, Faculty of Postgraduate Studies for Advanced Sciences, Beni-Suef University, Beni-Suef, 62511 Egypt; 2 Department of Microbiology and Immunology, Faculty of Pharmacy, Beni-Suef University, Beni-Suef, 62514 Egypt; 3 Department of Pharmaceutics and Industrial Pharmacy, Faculty of Pharmacy, Beni-Suef University, Beni-Suef, 62514 Egypt; 4 Pharmacology Department, Faculty of Veterinary Medicine, Beni-Suef University, Beni-Suef, 62511 Egypt

**Keywords:** Oleuropein, Hyalurosomes, Diabetic foot ulcers, Antioxidant, Anti-inflammatory, *TGFβ1*, Biochemistry, Biotechnology, Diseases, Drug discovery, Medical research

## Abstract

Diabetic foot ulcers (DFUs) are among the most severe and challenging complications associated with diabetes mellitus, primarily due to chronic inflammation, oxidative stress, and impaired tissue regeneration. To develop and evaluate oleuropein-loaded hyalurosomes (OLE-HLs) as a unique topical nanocarrier system for wound healing using an experimental diabetic rat model. OLE-HLs were synthesized using a modified thin-film hydration method, and the nano-formulation was characterized by measuring the size and potential, followed by TEM, encapsulation efficiency, FTIR, in vitro release studies, and cell cytotoxicity assay. The wound-healing efficacy was evaluated in vitro, using scratch assay and in vivo, via studying the wound closure rate, oxidative stress markers, pro-inflammatory cytokines, *TGFβ1* gene expression, and histopathological alterations, complemented by immunohistochemical analysis. OLE-HLs exhibited a mean particle size of 254.64 ± 9.84 nm, a zeta potential of − 25.12 ± 2.18 mV, and an encapsulation efficiency of 89.61 ± 1.82%, while TEM revealed spherical, uniformly sized, and well-dispersed vesicles, confirming stability and homogeneity. FTIR analysis verified successful oleuropein encapsulation and compatibility within the hyalurosomal system. The formulation displayed a biphasic release profile, with 26.47 ± 0.86% released in the first hour and 76.72 ± 2.45% within 24 h. Cytotoxicity assays showed enhanced bioactivity (IC_50_: 269.63 µg/mL) compared with free OLE (644.81 µg/mL). The scratching assay model was validated by the in vivo model, where OLE-HLs significantly promoted wound healing by reducing oxidative stress, as shown by increased GSH and GST levels and decreased MPO activity. It also suppressed inflammatory mediators (IL-6, IL-17, TNF-α, MMP-13, ADAMTS-5) and enhanced TIMP-3 expression. Additionally, regulation of TGFβ1 expression and improved histopathological and immunohistochemical features collectively supported better tissue repair. These data suggest that OLE-HLs notably promote diabetic foot ulcer healing by modulating inflammation, oxidative stress, and cellular regeneration, thereby warranting further clinical investigation.

**Supplementary Information:**

The online version contains supplementary material available at 10.1038/s41598-026-42804-5.

## Introduction

Diabetic foot ulcers (DFUs) are among the most prevalent and serious complications of diabetes mellitus, affecting approximately 6.4% of patients with diabetes globally^1,2^. These chronic wounds are driven by persistent oxidative stress, chronic inflammation, and impaired angiogenesis, often leading to delayed healing, infection, and lower-limb amputation, contributing to rising healthcare costs and poorer quality of life among these individuals^1^. Cytokines and growth factors play key roles in wound healing by regulating inflammation, tissue regeneration, and angiogenesis. Pro‑inflammatory cytokines like IL‑6 and TNF‑α mediate early responses, while anti‑inflammatory cytokines such as IL‑10 promote healing^3^. Growth factors like EGF, VEGF, PDGF, and TGF-β1 stimulate cell migration, ECM synthesis, and blood vessel formation, aiding tissue repair. These molecular signals ensure effective wound closure and tissue remodeling^4^.

Current treatments for DFUs are frequently inadequate, particularly in deep or exudative wounds, and they are often associated with prolonged healing times and adverse effects. Subsequently, there has been a growing interest in natural bioactive compounds known for their anti-inflammatory and regenerative properties^5^. Oleuropein (OLE), a major phytochemical found in olive leaf extract, possesses multiple bioactivities, including antioxidant, anti-inflammatory, and antimicrobial properties; however, its clinical use remains limited owing to its chemical instability, poor cellular permeability, rapid metabolism, and low bioavailability^6,7^.

Nanotechnology-based drug delivery systems provide novel approaches by enhancing drug solubility, physicochemical stability, bioavailability, prolonged drug half-life, and targeted delivery^8^. Phospholipid vesicles, particularly hyalurosomes (HLs), phospholipid-based nanovesicles modified with hyaluronic acid, are highly suitable for topical applications due to their structural similarity to biological membranes, favorable safety profile, and capacity to encapsulate both lipophilic and hydrophilic substances. In addition, HLs provide enhanced biocompatibility, gelling properties, improved skin retention, and superior penetration into deeper tissues^9,10^. Oleuropein-loaded hyalurosomes (OLE-HLs) combine the therapeutic efficacy of OLE with the structural and delivery advantages of HLs, offering a unique formulation for wound healing. To the best of our knowledge, no prior studies have explored the wound-healing potential of OLE-HLs gel in diabetic rats. Thus, this research sought to create an innovative OLE-HLs-based formulation, demonstrating a promise as a robust approach for effective wound healing.

## Materials and methods

### Materials

Oleuropein (OLE), cholesterol, sodium deoxycholate (SDC), lecithin, sodium carboxymethylcellulose (NaCMC), and streptozotocin (STZ) were purchased from Sigma-Aldrich (St. Louis, MO, USA). Chloroform and ethanol (HPLC grade) were received from Supelco Analytical Products (Germany). All additional reagents used in this work were of analytical grade.

### Preparation of OLE-HLs nano-formulation

OLE-HLs were synthesized by hydrating a thin lipid film method, as previously described^10^, with minor modifications. In brief, OLE (0.08 g) was dissolved in 5 mL of ethanol, and this solution was mixed with accurately weighed amounts of cholesterol (0.04 g), sodium deoxycholate (SDC) (0.078 g), and lecithin (0.4 g), all of which were solubilized in 10 mL of chloroform in an Erlenmeyer flask. The organic solvent was then evaporated using a rotary evaporator (Rotavapor, Heidolph VV 2000, Burladingen, Germany) at 100 rpm to form a thin lipid film. The resulting film was hydrated with an aqueous solution of 0.012 g of hyaluronic acid in 6 mL of phosphate-buffered saline (PBS), pH 7.4, for 1 h while stirring at ambient temperature. The dispersion was sonicated for 15 min using an ultrasonic cleaner (Ney, USA) to generate unilamellar vesicles and reduce the particle size. The vesicular dispersion was left to mature overnight at 4 °C before further analysis.

### Characterization and optimization of OLE-HLs nano-formulation

#### Size and potential determination

The average size, PDI, and surface charge of OLE-HLs were characterized by dynamic light scattering (DLS) on a Malvern Nano ZS90 (Malvern Instruments, UK). Before analysis, the dispersions were diluted 1:10 with deionized water. All measurements were performed at 25°C in triplicate (n = 3)^11^.

#### Morphological analysis

The morphology and surface properties of OLE-HLs were analyzed using transmission electron microscopy (TEM) on a JEM-2100 instrument (JEOL Ltd., Tokyo, Japan), operated at an accelerating voltage of 200 kV. A small drop of freshly prepared OLE-HLs was placed on a carbon-coated copper grid, which was then negatively stained with a 1% w/v solution of phosphotungstic acid. Excess stain was carefully blotted off with filter paper, and the grid was left to air dry for 10 min before examination^12^.

#### Evaluation of encapsulation efficiency

The encapsulation efficiency (EE%) of OLE in OLE-HLs was determined indirectly by measuring the unencapsulated OLE in the supernatant. The formulations were subjected to centrifugation at 15,000 rpm for 15 min at 4°C using a refrigerated centrifuge (SIGMA 3-30K, Germany)^13^. Free OLE was quantified using UV–VIS spectrophotometry at 280 nm^14^. All measurements were performed 3 times (n = 3), and EE% was calculated according to Eq. (1)

1 $$Encapsulation\;efficiency\user2{ } \left( \% \right) = \frac{Total\;amount \;of\;OLE - amount \;of\;Free\; OLE}{{Total \;amount\;of\;OLE}} \times 100$$

#### Fourier-transform infrared (FTIR) study

FTIR spectroscopy was utilized to evaluate molecular interactions between OLE and the hyalurosomes (HLs) matrix and to confirm characteristic functional groups, as described for OLE-loaded nanocarriers^15^. Dried samples of OLE and OLE-HLs were gently pressed onto a diamond attenuated total reflectance (ATR) crystal and scanned over 4000–400 cm^−1^ at 25 °C, recorded at 4 cm^−1^ resolution, and averaged over 64 scans using a Vertex 70 FTIR spectrometer (Bruker, Germany). Background spectra were recorded prior to each measurement. Spectral data were analyzed using OPUS software to detect peak shifts or intensity changes indicative of hydrogen bonding or other physicochemical interactions between OLE and the nanocarrier matrix.

#### In vitro release study of OLE

The release kinetics of OLE from OLE-HLs were assessed using a modified dialysis method^11^. A nano-dispersion corresponding to 3 mg of OLE was placed into glass cylinders sealed with presoaked cellophane semipermeable membranes with a molecular weight cut-off of 12–14 kDa and immersed in 200 mL of Sorensen’s phosphate buffer (pH 5.5) at 37°C, with continuous shaking at 150 rpm to maintain sink conditions. At specified time points (1, 2, 4, 6, 20, and 24 h), 2 mL samples were withdrawn and replaced with fresh buffer to maintain a consistent volume. OLE concentrations were measured using UV–VIS spectrophotometry at 280 nm^14^. Free OLE (unencapsulated) was evaluated under identical conditions. All experiments were performed three times, and the data are expressed as mean ± standard deviation (SD).

### In vitro cytotoxicity study

Cell viability was assessed using the MTT assay, which measures the mitochondrial-dependent reduction of yellow MTT (3-(4,5-dimethylthiazol-2-yl)-2,5-diphenyl tetrazolium bromide) to purple formazan^16^.

#### Cells and samples

Cytotoxicity of OLE and OLE-HLs was tested in human skin fibroblast (HSF) cells from ATCC, cultured in Dulbecco’s Modified Eagle Medium (DMEM) with 5% fetal bovine serum, 1% L-glutamine, and 1% antibiotic–antimycotic solution. Cells were maintained at 37 °C in a 5% CO_2_ Sartorius Stedium incubator.

#### MTT assay

HSF cells were maintained for 10 days before seeding into 96-well plates at a density of 1 × 10^3^ cells per well. Following a 24-h incubation, cells were subjected to different doses of OLE and OLE-HLs (15.62–1000 µg/mL) for a 48-h period, with untreated samples used as the negative control. Subsequently, each well received 20 μL of MTT solution (0.5 mg/mL) and was incubated for 4 h. The resulting formazan crystals were subsequently dissolved overnight in 200 μL of 10% SDS prepared in 0.01 M HCl at 37 °C. Absorbance was measured at 595 nm with 620 nm as reference using a Bio-Rad 3350 microplate reader (Hercules, CA, USA), and cell viability and cytotoxicity were calculated relative to control according to Eq. (2):

2 $${\mathrm{Viability}} = \frac{Absorbance\; of\;treated\;cells}{{absorbance\;of\;control\;cells}} \times 100$$

$$Cytotoxicity=100- viability$$

IC₃₀ and IC_50_ values were calculated using GraphPad Prism software (GraphPad Software Inc., San Diego, CA, USA). All assessments were repeated three times, and results are given as mean ± SD^17,18^.

### In vitro wound healing assay (Scratch assay)

Cell migration, a key process in tissue repair, was evaluated in HSF cells using a scratch wound assay^19^. HSF cells were seeded at a density of 5 × 10^5^ cells/well in 6-well plates and incubated overnight at 37 °C in a humidified 5% CO_2_ atmosphere to establish confluent monolayers. A straight linear wound was created through the adherent cell layer using a sterile 10 µL pipette tip. Detached cells were eliminated by washing twice with PBS. Subsequently, 3 mL of low-serum medium (DMEM + 1% FBS) with OLE and OLE-HLs was added at IC₃₀ concentrations determined from the MTT assay. Cell migration into the wound area was monitored at 0, 24, 48, and 72 h using an inverted microscope (ZEISS Axio Vert.A1) equipped with ZEN Blue software. Wound closure was quantified by measuring the residual width of the wound using the captured^20^.

### Preparation and characterization of transdermal gels

#### Formulation of transdermal-based gel

Topical nano-hybrid gels were created by incorporating either OLE-HLs or free OLE into a 3% w/w sodium carboxymethylcellulose (NaCMC) gel base. Specifically, 0.018 g of NaCMC was gradually added to each formulation, while maintaining continuous magnetic stirring to achieve a final oleuropein concentration of 2.5% w/w. The gels were allowed to hydrate overnight at 4 °C and were then evaluated for homogeneity, spreadability, and rheological behavior, as previously described^21,22^.

#### pH measurement

The pH of each hydrogel was determined using a calibrated digital pH meter (Jenway 3510, Cole-Parmer, UK). The electrode was immersed directly into the gel and allowed to equilibrate for 1 min^23^.

#### Spreadability

Approximately 0.5 ± 0.05 g of the gel sample was immersed on a 5 cm watch glass, overlaid with a second glass, and left for 5 min. The diameter of the spread gel was measured to assess its spreadability^24^.

#### Homogeneity

Gel samples from various regions of each formulation were dissolved in PBS pH 5.5 at a ratio of 1 g to 9 mL (1:9 w/v) and analyzed spectrophotometrically at 280 nm to evaluate the uniformity of drug distribution^22^.

#### Rheological properties determination

Hydrogel rheology was measured using a cone-and-plate viscometer (DV2T, AMETEK Brookfield, USA, spindle CP52). A 0.5 g sample was tested at 25 ± 2 °C, over a shear rate range of 20–400 s^−1^^25^.

### In vivo study and experimental design

Twenty-five adult male Wistar rats (145–170 g) were obtained from the Farm Lab Animal Facility (Helwan, Cairo, Egypt) and handled according to the Institutional Animal Care and Use Committee (IACUC), Faculty of Postgraduate Studies for Advanced Sciences, Beni-Suef University, Egypt, and were approved by the committee (Approval No. PSAS-BSU-HAREC-025–08-07). Following a 15-day acclimation, rats were housed five per cage under controlled conditions (25 ± 2 °C, 60% humidity) with a 12-h light/dark cycle and free access to food and water. Animals were then randomly assigned to five experimental groups for diabetes induction and subsequent analyses. This study was conducted and is reported in accordance with the ARRIVE guidelines (Animal Research: Reporting of In Vivo Experiment).

#### Induction of diabetes mellitus and wound creation

Rats were rendered diabetic by feeding a high-fat, high-carbohydrate diet for 21 days to promote insulin resistance. Subsequently, a single low-dose intraperitoneal (IP) injection of streptozotocin (STZ; 10 mg/kg body weight) was given. STZ was freshly prepared in citrate buffer pH 4.5 at 20 mg/mL and sterilized by a 0.2 µm Millipore syringe filter before administration^26^. Blood glucose levels were measured from tail vein samples 48 h post-injection using a commercial glucometer (GlucoDr), and rats exhibiting glucose levels > 200 mg/dL were considered diabetic and included in subsequent experiments^27^. Fasting blood glucose levels were measured weekly throughout the experimental period to assess the stability of hyperglycemia. Only animals with consistently elevated glucose levels were included in the study. Wound induction was carried out as previously described by Correa et al.^28^. Diabetic rats were anesthetized via IP injection of xylazine (5 mg/kg) and ketamine (90 mg/kg), following the optimized protocol of Sotoudeh et al.^29^. A round, full-thickness excisional skin wound (~ 1 cm in diameter) was created on the front right leg of the rats using sterile Westcott scissors and forceps. The wounds were cleaned with a sterile cotton swab moistened with 0.9% saline and left undressed for the entire duration of the study^30^.

#### Study design and treatments

The animals were randomly allocated into five groups (n = 5 per group) as follows:

- *Normal Control group (NC)*: Non-diabetic rats with excisional wounds, left untreated.
- *Diabetic foot ulcer group (DFU)*: Diabetic rats with excisional wounds, left untreated.
- *Oleuropein gel group (OLE)*: Diabetic rats treated topically with 0.5 g/day of free OLE hydrogel.
- *Oleuropein-hyalurosomes group (OLE-HLs)*: Diabetic rats treated topically with 0.5 g/day of 2.5% w/w OLE-HLs hydrogel.
- *Fucidin group (Fucidin)*: Diabetic rats treated topically with 0.5 g/day of Fucidin cream (fusidic acid, FA; LEO Pharma, Denmark).

Topical treatments were initiated on the day of wound induction (Day 0) and applied once daily at 12:00 PM for a period of 14 consecutive days. Before treatment application, the Cutaneous wounds were gently cleaned with sterile gauze soaked in 0.9% sterile saline solution. A 0.5 g dose of the respective gel treatment was then applied to the wounds. The concentration of 2.5% w/w OLE-HLs hydrogel was selected based on preliminary formulation optimization and prior literature, ensuring effective OLE loading for wound healing while maintaining suitable rheological properties for topical application and minimizing potential irritation or toxicity. The wounds were left exposed throughout the study duration. Wound healing was assessed through daily monitoring, and the wound areas were measured systematically to evaluate the efficacy of the treatments.

#### Macroscopic analysis

Under sterile conditions, the wound area was measured using a transparent millimeter scale in the excisional model on the 4th, 7th, 10th, and 14th days. The percentage of wound healing was calculated using the following Eq. (3)^27^:

3 $$\text{Wound closure }(\mathrm{\%})=\frac{\text{Initial wound area}-\text{Wound area on }{N}^{TH}\mathrm{day}}{\text{Initial wound area}}\times 100$$

#### Samples preparation

At the end of the study, all animals were humanely euthanized under deep anesthesia via IP injection of ketamine-xylazine (0.1 mL/100 g body weight), following institutional and ethical guidelines^29^. Blood samples were collected from the tail vein, allowed to clot at room temperature, and then centrifuged at 3000 rpm for 30 min. The clear, non-hemolyzed serum was carefully separated, aliquoted into three portions, and stored at − 20 °C for future biochemical analyses. For plasma glucose determination, blood was drawn into sodium fluoride-containing tubes to inhibit glycolysis, followed by centrifugation to separate plasma. Additionally, for glycated hemoglobin (HbA1c) analysis, blood was collected into EDTA tubes to preserve hemoglobin integrity.

### Biochemical analyses

#### Metabolic profiling

Random plasma glucose and glycated hemoglobin (HbA1c) levels were determined using the ichrom fluorescence immunoassay system (Boditech Med Inc., Korea), according to the manufacturer’s instructions. Serum insulin levels were quantified using a rat-specific ELISA kit (Abcam, UK; cat. no. ab277390). Serum lipid profiles were determined using standard enzymatic colorimetric methods kits (SPINREACT, Spain). LDL-C was calculated using the Friedewald Equation (4)^31^:

4 $$LDL-C =TC- [HDL-C + (TG\div 5)]$$

where LDL-C is low-density lipoprotein cholesterol, TC is total cholesterol, HDL-C is high-density lipoprotein cholesterol, and TG is triglycerides.

#### Oxidative stress biomarkers

Myeloperoxidase (MPO) activity was assessed using a rat-specific sandwich ELISA kit (BioVision, USA; cat. no. E4581-100)^32^. Reduced glutathione (GSH) levels were measured via a colorimetric enzymatic cycling method (BioVision; cat. no. K464-100)^33^. Glutathione S-transferase Yb1 (GST Yb1) was quantified using an ELISA kit (Creative Diagnostics, USA; cat. no. DEIA8527)^34^.

#### Inflammatory and ECM-degrading markers

Tissue levels of pro-inflammatory cytokines TNF-α, IL-6, and IL-17 were measured using ELISA kits: TNF-α (BioLegend, USA), IL-6 (BioVision; cat. no. K4145-100), and IL-17 (Wuhan Fine Biotech, China; cat. no. ER0035), following manufacturer guidelines.

Protein expression levels of matrix metalloproteinase-13 (MMP-13), A Disintegrin and Metalloproteinase with Thrombospondin Motifs 5(ADAMTS-5), and tissue inhibitor of metalloproteinases-3 (TIMP-3) were assessed by western blotting. Primary antibodies used were MMP-13 (Santa Cruz Biotechnology; clone C-3, cat. no. sc-515284), ADAMTS-5 (Abcam; cat. no. ab41037), and TIMP-3 (Santa Cruz Biotechnology; clone B-2, cat. no. sc-373839)^35^.

#### Molecular investigations

Total RNA was isolated from tissue samples using the Direct-zol RNA Miniprep Plus Kit (Research, USA). Only RNA with A260/A280 ratios of 1.8–2.0 was used. Quantitative RT-PCR was performed with the SuperScript IV One-Step RT-PCR System (Thermo Fisher Scientific, USA) on a StepOne Real-Time PCR platform. Each 50 µL reaction contained master mix, primers (Table 1), and 10 µL RNA. Thermal cycling conditions were 55°C for 10 min, 95°C for 2 min, followed by 40 cycles of 95°C for 10 s, 55°C for 10 s, and 72°C for 30 s, with a final 72°C extension for 5 min. All reactions were performed in triplicate. Relative *TGFβ1* levels were calculated using the 2^–ΔΔCt^ method with *GAPDH*^36^.

**Table 1:** Primer sequences for real-time PCR.

	Accession no	Forward sequence	Reverse sequence
*TGFβ1*	NM_021578.2	GACTCTCCACCTGCAAGACC	GGACTGGCGAGCCTTAGTTT
*GAPDH*	XM_017592435.1	CACCCTGTTGCTGTAGCCATATTC	GACATCAAGAAGGTGGTGAAGCAG

### Histopathological examination

Freshly excised full-thickness skin samples (including wound margins) were collected and processed using the standard paraffin-embedding technique, following the protocol of Bancroft and Gamble^37^. Tissues were fixed in 10% neutral-buffered formalin for 48 h, dehydrated through graded ethanol (70% for 1.5 h, 90% for 1.5 h, and 100% for 3 h), cleared in xylene (4 h), and infiltrated with paraffin wax at 56 °C through three sequential changes (1 h each). Samples were then embedded in paraffin blocks at 58 °C, sectioned at 3–5 µm thickness using a rotary microtome, and mounted on clean glass slides. Sections were stained with hematoxylin and eosin (H&E) and cover slipped using Distrene–Plasticiser–Xylene (DPX) mounting medium. Histological assessments were performed under a light microscope at × 200 and × 400 magnifications, and representative micrographs were captured to evaluate tissue morphology and wound healing progression.

### Immunohistochemistry (IHC) for NF-κB

Immunohistochemistry for Nuclear Factor kappa-light-chain-enhancer of activated B cells (NF-κB) was performed on 4 µm paraffin-embedded sections using a modified avidin–biotin complex (ABC) immunoperoxidase method as previously described^38^. Paraffin-embedded tissues were deparaffinized, rehydrated, and subjected to antigen retrieval by microwave heating in citrate buffer pH 6.0 for 5 min at 700 W. Endogenous peroxidase activity was quenched, followed by protein blocking to reduce non-specific binding. Sections were incubated with the primary NF-κB p65/RelA antibody for 30 min at room temperature, followed by a biotinylated secondary antibody and streptavidin-HRP. Staining was visualized with 3,3′-diaminobenzidine (DAB), a substrate–chromogen solution (5–10 min). Sections were counterstained with Harris’s hematoxylin, dehydrated through graded alcohols, cleared in xylene, and mounted with DPX. All stained slides were examined using a light microscope, and photomicrographs were captured with a LEICA DFC290 HD digital camera (Switzerland).

### Statistical evaluation

All results were presented as mean values accompanied by the standard error of the mean (SE) to describe central tendency and variation. Statistical evaluation was conducted using SPSS software (version 22.0; IBM Corp., Armonk, NY, USA). Differences among multiple groups were analyzed by one-way ANOVA, followed by Duncan’s multiple range test to determine pairwise differences. A significance level of *p* < 0.05 was applied. Prior to ANOVA, the assumptions of normal distribution and homogeneity of variance were verified through the Shapiro–Wilk and Levene’s tests.

## Results and discussion

### Physicochemical characterization of OLE-HLs nano-formulation

#### Particle size, polydispersity, and zeta potential analysis

**Fig. 1:**
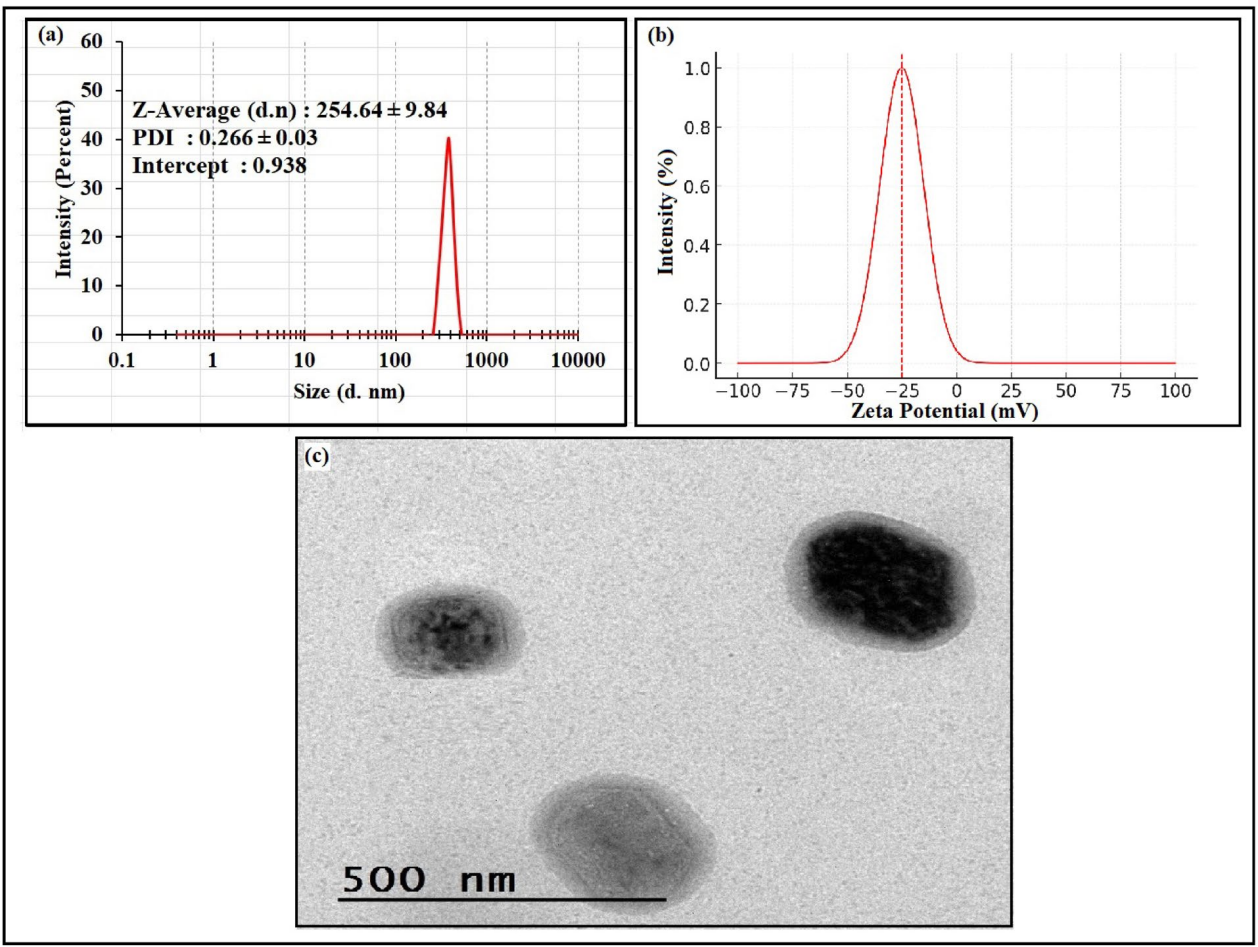
(**a**) Particle size, (**b**) zeta potential, and (**c**) TEM of OLE-HLs.

Particle size plays a fundamental role in the design of nanocarrier-based drug delivery systems, as it directly influences formulation stability, skin permeability, and biodistribution profiles^39^. In this study, DLS analysis revealed that the optimized OLE-HLs nano-formulation exhibited an average particle size of 254.64 ± 9.84 nm (Fig. 1a), confirming their classification within the nanometric range (< 1000 nm)^40^. The polydispersity index (PDI) of the OLE-HLs formulation was 0.266 ± 0.03, indicating a relatively narrow particle size distribution and good uniformity. PDI values below 0.5 suggest acceptable homogeneity in nanoscale systems, which supports the reproducibility, physical stability, and suitability of the OLE-HLs nano-vesicles for topical delivery applications^41^. Nanosystems with diameters below 500 nm are particularly advantageous for topical delivery^39^. In line with this, the OLE-HLs formulation significantly enhanced the topical bioavailability of oleuropein by promoting skin permeation, improving dermal absorption, and increasing local therapeutic efficacy^42^.

Zeta potential is a critical physicochemical parameter that reflects nanoparticle surface charge and reliably indicates colloidal stability in nanosuspensions^42^. In our study, OLE-HLs nanovesicles exhibited a high negative zeta potential of − 25.12 ± 2.18 mV (Fig. 1b), indicating strong electrostatic repulsion and excellent colloidal stability^43^. This substantial surface minimizes vesicle aggregation and preserves nano-system integrity during storage and topical application, which are considered key factors for maintaining formulation performance^44^. Consistent with previous findings, absolute zeta potential values exceeding ± 20 mV are generally considered predictive of physically stable nano-dispersions, reinforcing the suitability of OLE-HLs for topical drug delivery^42,44^.

#### Transmission electron microscope (TEM)

TEM was employed to investigate the morphological characteristics of the optimized OLE-HLs formulation^45^. The micrographs revealed that the vesicles were spherical, uniformly sized, well-dispersed, and exhibited smooth surfaces with no signs of aggregation, indicating good physical stability and confirming the suitability of the synthesized formulation (Fig. 1c). The observed morphology aligns with previous reports, which describe hyalurosomal vesicles as spherical to slightly oval with small-diameter features, favorable for enhancing permeability across biological membranes, particularly in topical delivery systems^10^. The shape and surface properties of nanocarriers significantly influence their cellular uptake, tissue distribution, and therapeutic outcomes. Accordingly, the spherical structure of OLE-HLs is expected to enhance skin permeation and promote localized delivery of OLE, thereby improving its bioavailability and therapeutic effect^46^.

Additionally, TEM confirmed the vesicle size observed by DLS, validating the measurements obtained from the Malvern system^47^. However, size measurements obtained by TEM often differ from those recorded by DLS. The variation can be explained by the vacuum environment applied during TEM imaging and the fluid and electrokinetic effects intrinsic to light-scattering analysis. Whereas TEM focuses on one particle at a time, scattering techniques assess the overall size distribution of nearly 10^11^ particles, reporting a mean value after fitting^48^.

#### Encapsulation efficiency percentage (EE%)

The optimized OLE-HLs formulation exhibited a high encapsulation efficiency of 89.61 ± 1.82%, consistent with previous reports on similar nanocarriers^39,49^. This efficiency is attributed to the structural features of OLE, including hydrogen-bonding groups (–OH, –C = O) and its amphiphilic nature (i.e., moderate amphiphilicity), which favor its interaction with the hyalurosomes matrix^50,51^.

Hyalurosomes represent a valuable platform for topical drug delivery, given their efficiency in encapsulating substantial levels of therapeutic agents. Their dual aqueous and lipid environments enable efficient entrapment of OLE, with the hydrophilic glycoside moiety favoring incorporation into the aqueous core, while the aromatic rings facilitate partial interaction with the phospholipid bilayer^51^. The presence of hyaluronic acid further stabilizes the vesicles and enhances drug retention via hydrogen bonding, supporting sustained release and improved topical bioavailability^39^.

#### FTIR spectra analysis

**Fig. 2:**
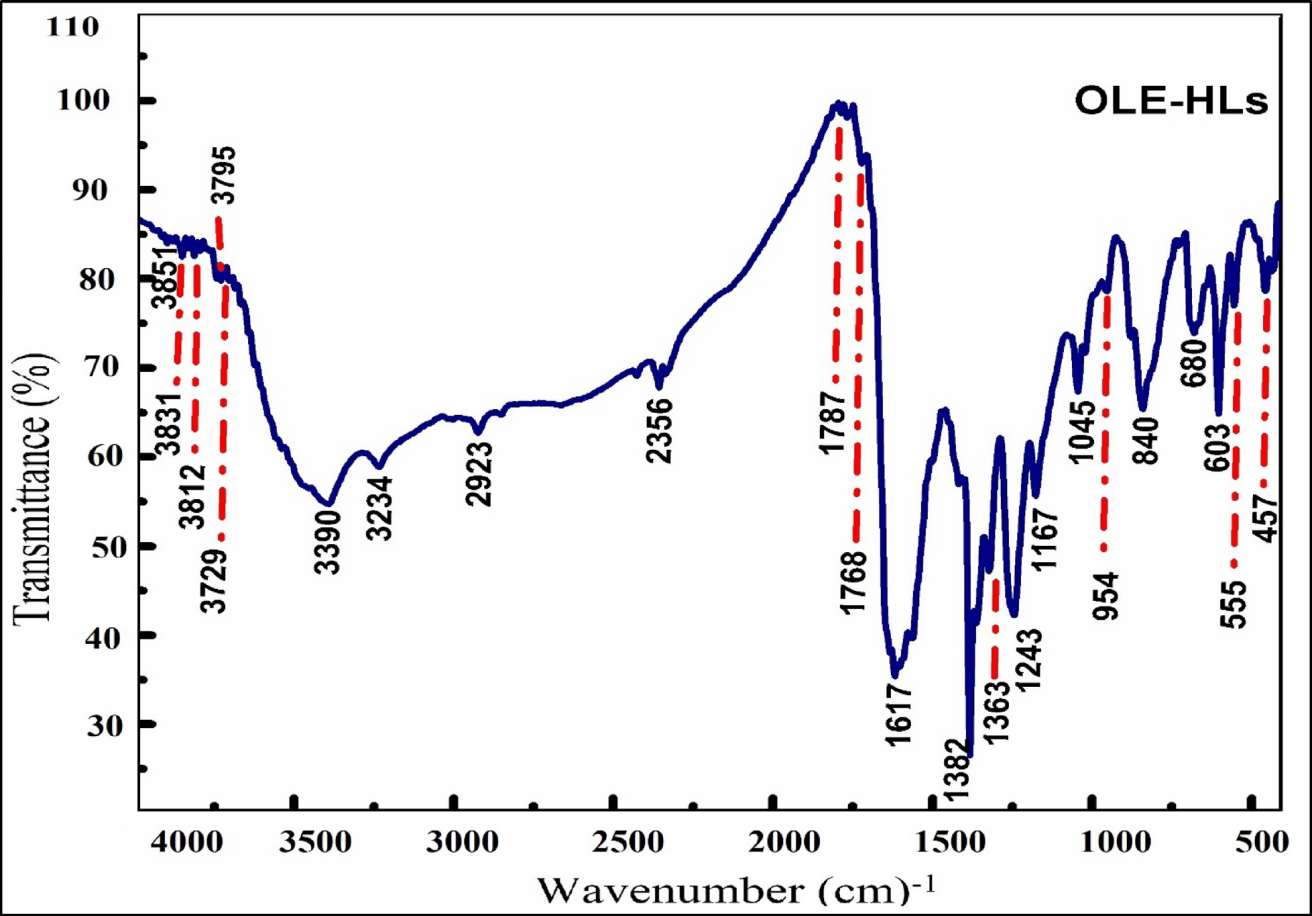
Fourier-transform infrared spectroscopy (FTIR) spectra of OLE-HLs nano-formulation.

FTIR spectroscopy was performed to assess potential molecular interactions and to confirm the successful entrapment of OLE within the hyalurosomes vesicles. The FTIR spectrum of pure OLE exhibited characteristic absorption bands at approximately 3390 cm^−1^ (O–H stretching), 1720–1750 cm^−1^ (C=O stretching of ester or carboxylic groups), 1615–1625 cm^−1^ (aromatic C=C stretching), and 1045–1065 cm^−1^ (C–O stretching). These peaks are consistent with previously reported spectra for crude OLE^52^. The spectra of OLE-HLs displayed the same characteristic bands with slight shifts and reduced intensities, indicating successful physical entrapment of OLE within the vesicles without evidence of chemical degradation (Fig. 2).

In addition, peaks attributed to lecithin were evident, including bands at 2923 and 2853 cm^−1^ (aliphatic C–H stretching), ~ 1740 cm^−1^ (ester C = O), 1240 cm^−1^ (P = O asymmetric stretching), and 1062 cm^−1^ (P–O–C stretching), which are consistent with phospholipid-based vesicles^53,54^. Cholesterol-specific bands were also observed at ~ 3400 cm^−1^ (O–H stretching), 2932–2899 cm^−1^ (aliphatic C–H stretching), 1464 cm^−1^ (CH_2_ bending), and ~ 1055 cm^−1^ (C–O stretching), confirming its incorporation into the lipid bilayer^54,55^. Sodium deoxycholate exhibited characteristic bile salt signals at ~ 3400 cm^−1^ (O–H stretching), 2930–2850 cm^−1^ (CH_2_/CH₃ stretching), 1610–1620 cm^−1^ (COO⁻ asymmetric stretching), and ~ 1450 cm^−1^ (CH_2_ bending)^56^. The presence of hyaluronic acid was confirmed by a broad O–H stretch around 3400 cm^−1^, a carboxylate band at 1600–1650 cm^−1^, and a polysaccharide-related C–O–C stretch at ~ 1038 cm^−1^^57^.

No new peaks or major shifts were observed in the spectra, indicating the chemical stability of the components and successful compatibility of OLE within the hyalurosome matrix (Fig. 2).

#### In-vitro release profile of OLE vs OLE-HLs drug release studies

The release profile of OLE from the optimized OLE-HLs formulation was evaluated using the dynamic dialysis method at pH 5.5, simulating the mildly acidic environment of human skin while maintaining vesicle integrity. Drug release from nanocarriers is influenced by several interdependent factors, including the physicochemical properties of the drug, the carrier’s architecture, and interactions with the release medium. In this formulation, OLE release is primarily governed by a combination of Fickian diffusion, matrix relaxation, and polymer swelling, resulting in a controlled and sustained release under physiologically relevant conditions^58,59^.

**Fig. 3:**
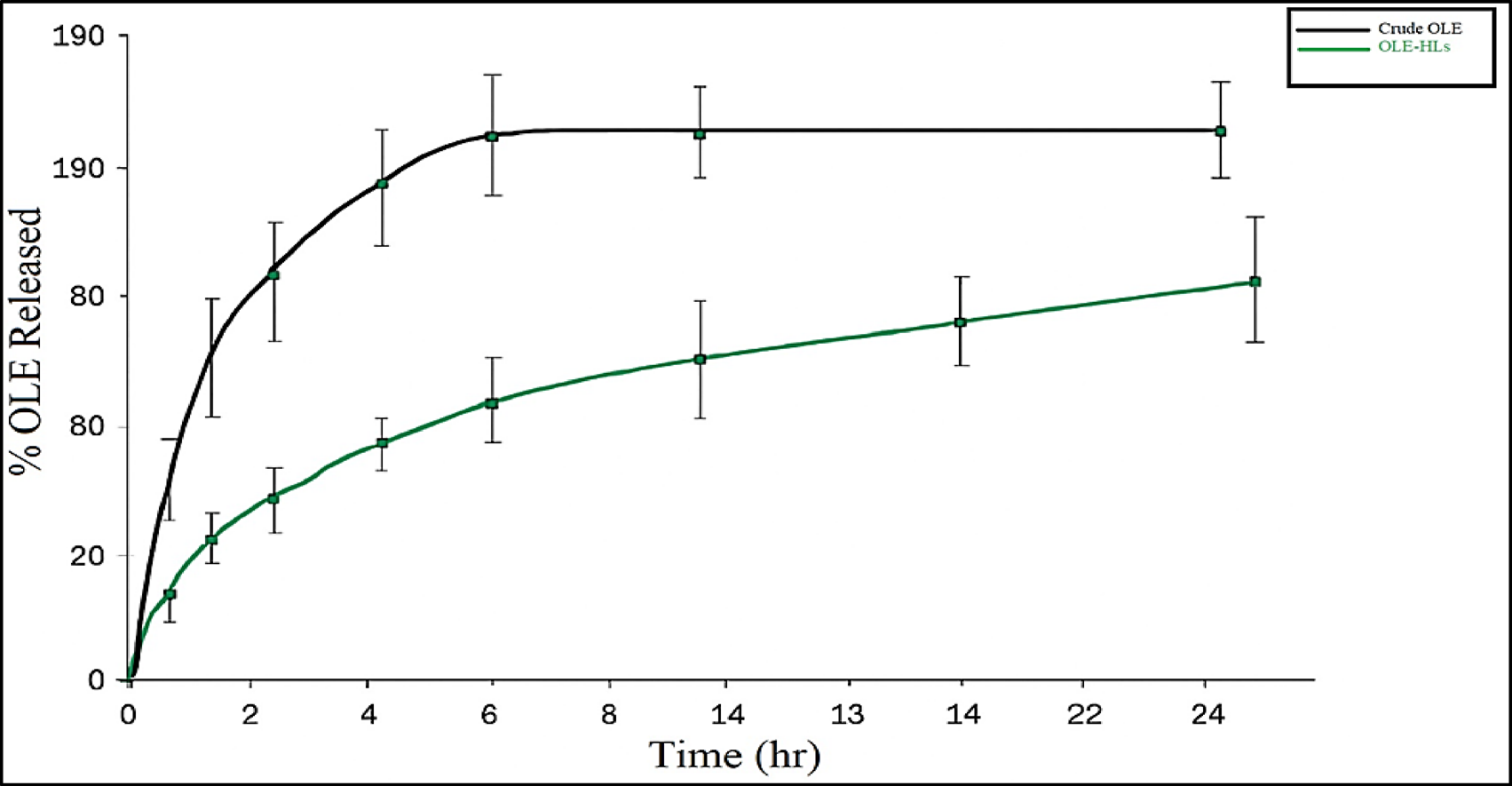
In vitro release of OLE from free OLE and OLE-HLs in PBS (pH 5.5, 37 °C).

As illustrated in Fig. 3, OLE-HLs exhibited a biphasic release profile, with an initial rapid release of 26.47 ± 0.86% during the first hour, which is attributed to surface-associated or loosely bound OLE, followed by a sustained release phase, reaching 76.72 ± 2.45% at 24 h. This behavior reflects the structural organization of hyalurosomes, where OLE is distributed between the aqueous core and the phospholipid bilayer. In contrast, free OLE demonstrated a burst release of 59.74 ± 1.16% within the first hour, with nearly a complete release (~ 100%) occurring within 8 h, owing to its rapid solubilization in the absence of a carrier matrix.

The sustained release from OLE-HLs can be attributed to several formulation-related factors, including their nanometric size, moderately negative zeta potential, and the amphiphilic architecture of the phospholipid bilayer, which collectively facilitate efficient drug entrapment and controlled diffusion. Moreover, the incorporation of hyaluronic acid, a key structural component of hyalurosomes, enhances mucoadhesion, skin retention, and barrier penetration, thereby prolonging drug release and improving dermal bioavailability^39,60^.

These findings are consistent with earlier reports on vesicular systems encapsulating oleuropein or similar polyphenols, which demonstrated biphasic release profiles governed by diffusion, matrix swelling, and polymer relaxation mechanisms^58,59,61^. Collectively, OLE-HLs demonstrate significant potential as a nanocarrier system for topical drug delivery, enabling prolonged release, enhanced skin targeting, and improved therapeutic outcomes in wound healing applications.

### Optimization of the stability of OLE-HLs gel

The prolonged stability of topical formulations is essential for maintaining therapeutic efficacy, usability, and patient compliance. In this study, a comparative evaluation was conducted between OLE-HLs and free OLE gels over three months of storage. The nanocarrier-based formulation demonstrated superior performance in terms of pH stability, homogeneity, spreadability, and rheological behavior (Table 2).

**Table 2:** Physicochemical characterization of OLE-HLs Gel versus free OLE Gel.

Parameter	OLE-HLs Gel	Free OLE Gel
Initial pH	5.85 ± 0.04	5.91 ± 0.03
pH after 3 months	5.76 ± 0.05	5.68 ± 0.06
Spreadability (cm)	6.8 ± 0.23	5.9 ± 0.32
Viscosity (cps)	13,950 ± 280	12,300 ± 310
Rheological behavior	Pseudoplastic (shear-thinning)	Pseudoplastic (shear-thinning)
Homogeneity	Excellent, no aggregation	Good, slight aggregation at room temp
Physical appearance	Smooth, stable, no phase separation	Slight thickening at 25°C

Data are expressed as mean ± SE.

#### PH stability

Both formulations maintained pH values within the physiologically acceptable dermal range (5.0–6.5) over three months of storage. Initially, the pH of the OLE-HLs gel was 5.85 ± 0.04, while that of the free OLE gel was 5.91 ± 0.03. No significant variation was observed over time (*p* > 0.05), though the OLE-HLs gel exhibited superior pH stability. This superior stability may be attributed to the encapsulation of OLE within the HLs, which protects the active compound from environmental degradation. These results are consistent with prior findings emphasizing the buffering role of nanocarriers in stabilizing pH-sensitive agents^39^.

#### Homogeneity

Both gels showed visual uniformity without phase separation or sedimentation. Microscopic analysis revealed uniform dispersion in both systems. However, minor aggregation was observed in the free gel after prolonged storage, whereas the OLE-HLs gel maintained stable particle distribution at ambient temperature. These observations reinforce the role of nanoencapsulation in enhancing colloidal stability and preventing drug crystallization or oxidation^62^.

#### Spreadability

Spreadability is a key rheological parameter that influences the ease of application, patient adherence, and the uniformity of drug distribution. The OLE-HLs gel demonstrated significantly greater spreadability (6.8 ± 0.2 cm) compared to the free OLE gel (5.9 ± 0.3 cm). This enhancement is attributed to the combined effects of hyaluronic acid’s viscoelastic and hydrophilic properties, along with the deformable structure of the nanocarriers, which decreases internal resistance and improves flow behavior. Enhanced spreadability facilitates better skin coverage and supports localized drug delivery, which is essential for effective wound therapy. This outcome concurs with previous evidence indicating that nanostructured gels improve topical performance^63,64^.

#### Rheological behavior

**Fig. 4:**
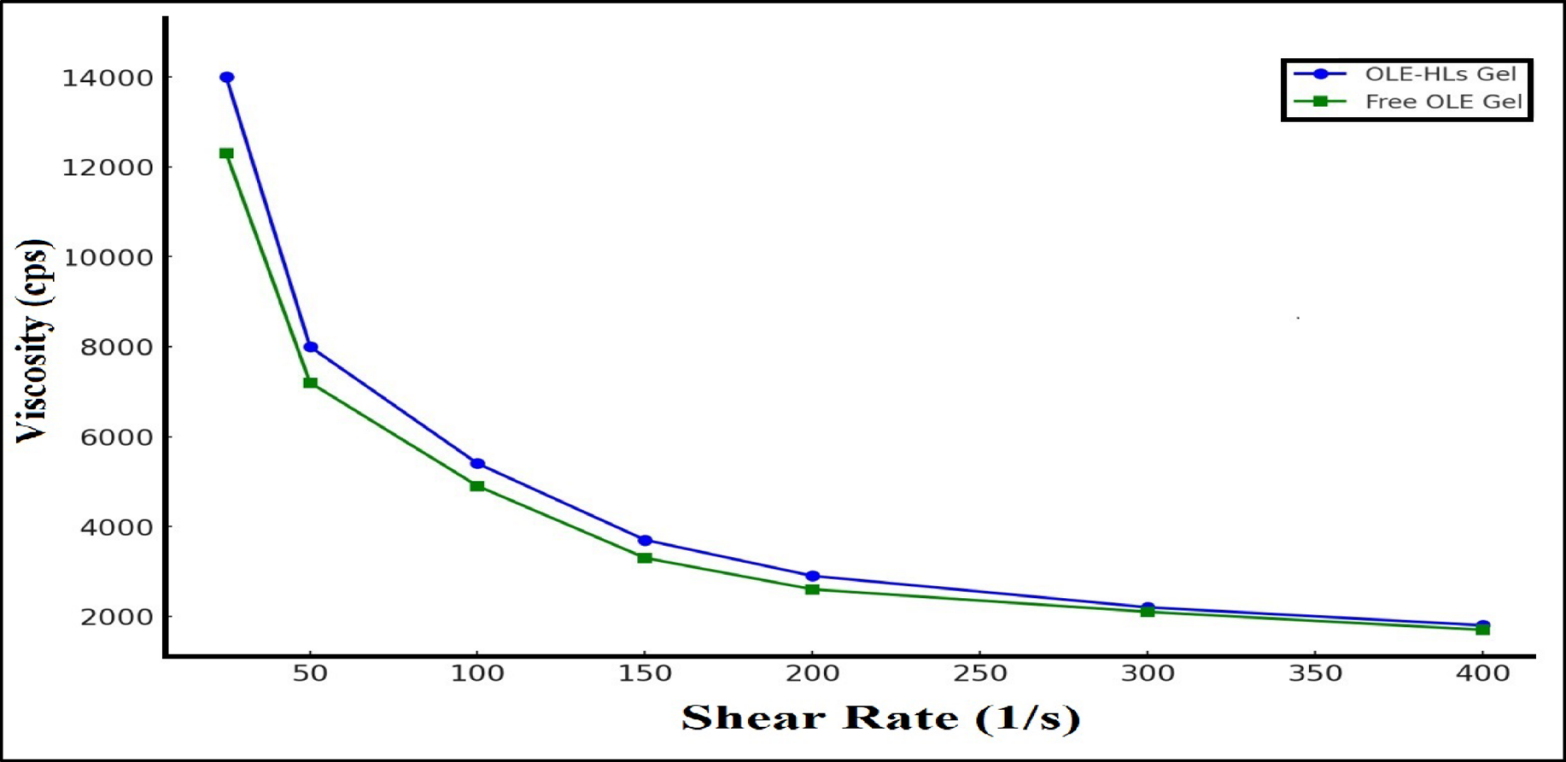
Rheological comparison of OLE-HLs and free OLE gels showing pseudoplastic behavior.

Both formulations demonstrated pseudoplastic (shear-thinning) behavior, showing reduced viscosity at higher shear rates, an important trait for topical gels. The OLE-HLs gel consistently showed higher viscosity values (13,950 ± 280 cps) across all shear rates compared to the free OLE gel (12,300 ± 310 cps), as illustrated in Fig. 4. This higher viscosity indicates superior structural integrity, possibly resulting from the interpenetrating network of nanocarriers and gel matrix. Enhanced rheological properties further support the suitability of the OLE-HLs gel for topical administration, especially in chronic wound settings where formulation retention is critical for therapeutic efficacy^65,66^.

### Cell cytotoxicity (IC_50_)

**Fig. 5:**
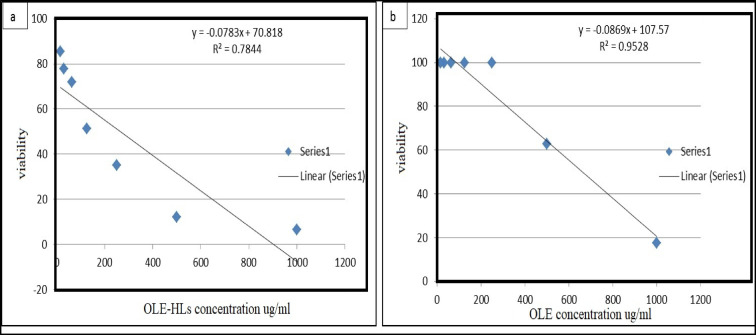
Concentration-dependent effects of OLE-HLs and free OLE on HSF cell viability.

The cytotoxicity of OLE and OLE-HLs was evaluated using the MTT assay on normal HSF cells. The free OLE solution exhibited excellent biocompatibility, maintaining nearly 100% cell viability at concentrations up to 250 µg/mL, with an IC_50_ of 644.82 µg/mL and IC₃₀ of 425.43 µg/mL. Conversely, the OLE-HLs formulation showed lower levels compared with pure OLE. IC_50_ was 269.60 µg/mL, and IC₃₀ was 69.31 µg/mL, and the reduction in cell viability was 6.5% at the highest tested concentration (1000 µg/mL), for OLE-HLs as shown in Fig. 5. These findings suggest that nanoencapsulation enhances cellular uptake and intracellular drug accumulation, indicating that the formulation improves delivery efficiency without inducing biologically relevant cytotoxic effects. However, OLE-HLs remained biocompatible at therapeutically relevant doses (< 62.5 µg/mL), supporting their potential use in topical wound healing applications where controlled release and cellular compatibility are critical. Similar observations have been reported in earlier studies^7,67^.

### Assessment of wound recovery process

Wound healing is a dynamic, multi-phase process designed to restore tissue integrity and function. It proceeds through four interrelated and overlapping stages: hemostasis, inflammation, proliferation, and remodeling. Each phase is orchestrated by complex cellular and molecular interactions involving clot formation, immune modulation, tissue regeneration, and extracellular matrix remodeling^68^.

OLE demonstrates pronounced antioxidant and anti-inflammatory effects, antimicrobial, and regenerative properties that enhance wound repair by promoting re-epithelialization, granulation tissue development, angiogenesis, and collagen production^69,70^.

#### In vitro wound healing

The wound-healing assay, also referred to as the scratch assay, is a straightforward and frequently employed in vitro method for the investigation of cell migration. It entails the formation of a “wound” (a cell-free space) in a confluent layer of cells and the subsequent observation of their migration to close the gap. This assay aids researchers in comprehending the function of cell movement in wound healing and other biological processes^71^.

**Fig. 6:**
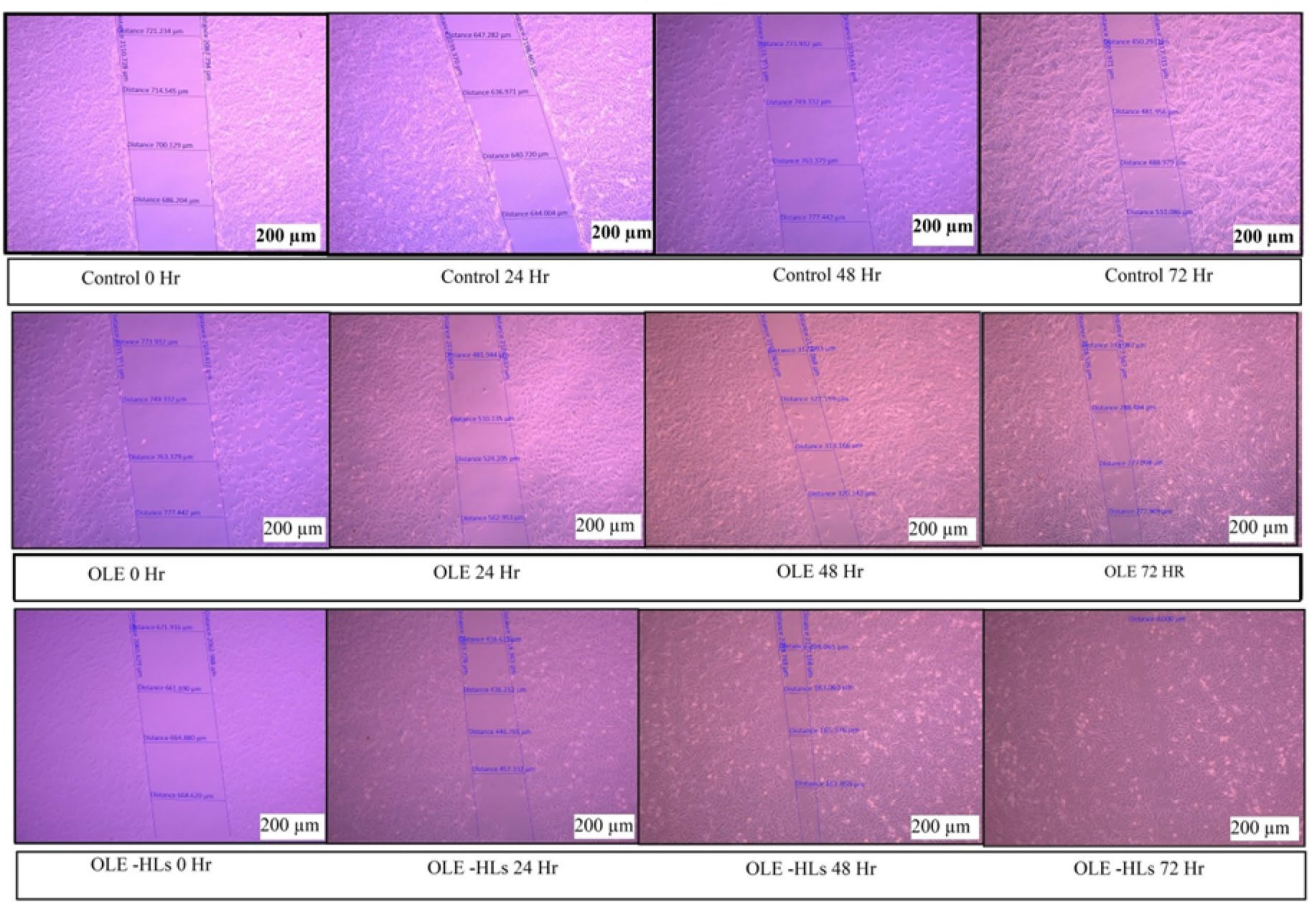
Microscopic images of scratch assay in Control, OLE, and OLE-HLs groups at 0–72 h (× 100).

**Fig. 7:**
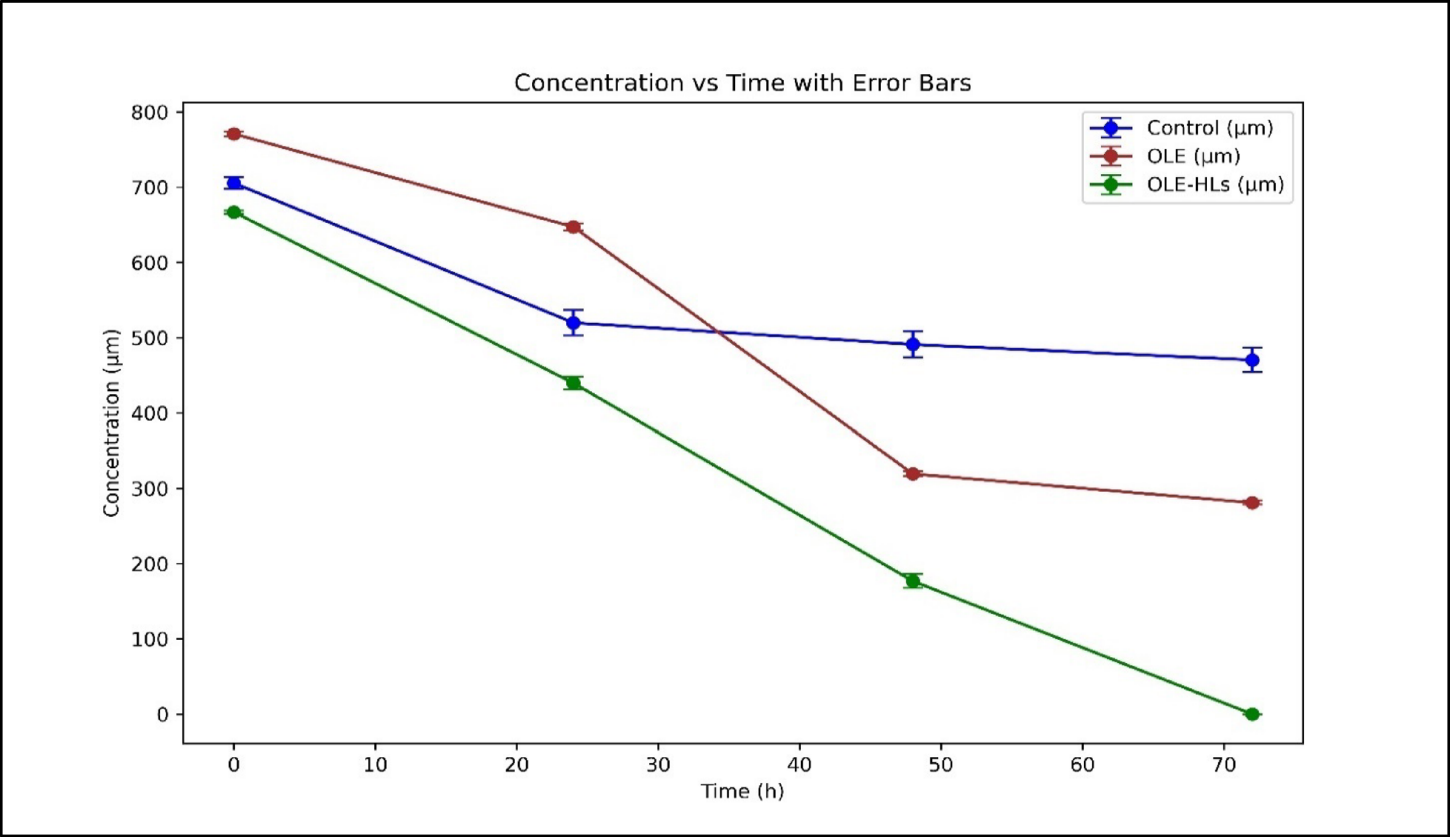
In vitro scratch assay showing wound closure (%) over 72 h in Control, OLE, and OLE-HLs groups*.*

In the current study, the scratch assay demonstrated significantly accelerated wound closure in the OLE-HLs group compared to pure OLE and control. At 24 h, wound closure reached 34.92% with OLE-HLs, versus 15.91% and 19.82% for free OLE and control, respectively. By 72 h, OLE-HLs achieved nearly complete closure (99.70%), while free OLE and control reached 63.84% and 32.16%. This superior performance is possibly due to the enhanced bioavailability and prolonged release of OLE via the hyalurosomal system, promoting effective cell migration and wound healing (Table 3; Figs. 6 and 7), which was consistent with several previous findings^7,39,72,73^.

**Table 3:** Results of scratch assay showing wound closure (in µm and Percentage) over time for OLE-HLs, OLE, and control groups.

Time (h)	Control (µm)	OLE (µm)	OLE-HLs (µm)	Control (%)	OLE (%)	OLE-HLs (%)
0	707.0 ± 7.8	769.3 ± 3.1	665.3 ± 2.2	0%	0%	0%
24	566.7 ± 16.9	647.0 ± 5.0	433.0 ± 8.2	19.8%	15.9%	34.9%
48	482.0 ± 17.5	325.3 ± 3.0	188.7 ± 9.3	31.8%	57.7%	71.6%
72	480.3 ± 16.1	278.3 ± 2.6	0.0 ± 0.0	32.1%	63.8%	100%

Data expressed as mean ± SE.

#### In vivo (Macroscopic) evaluation of wound healing

**Fig. 8:**
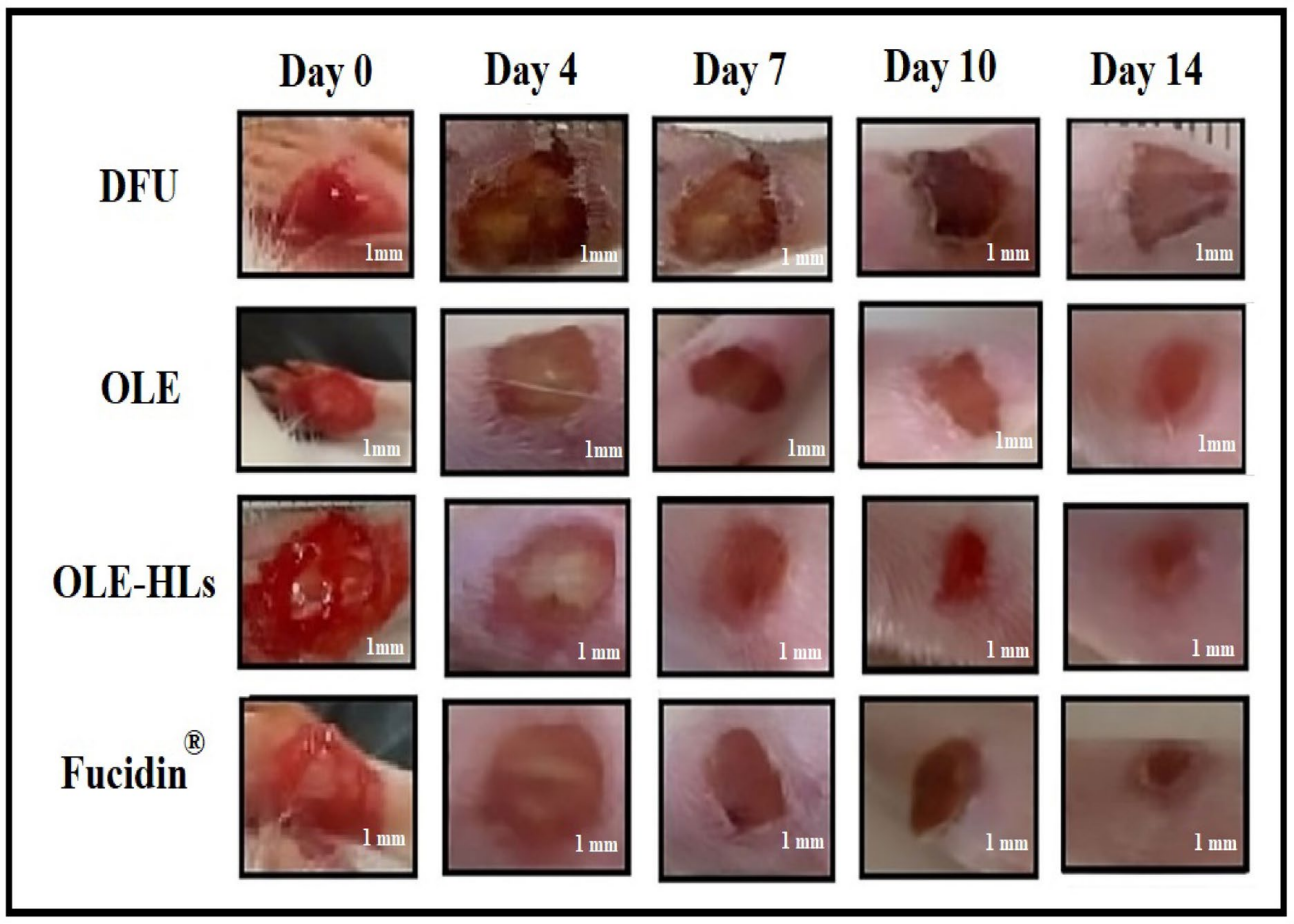
Representative wound images of diabetic rats at days 0, 4, 7, 10, and 14 under various treatments.

**Fig. 9:**
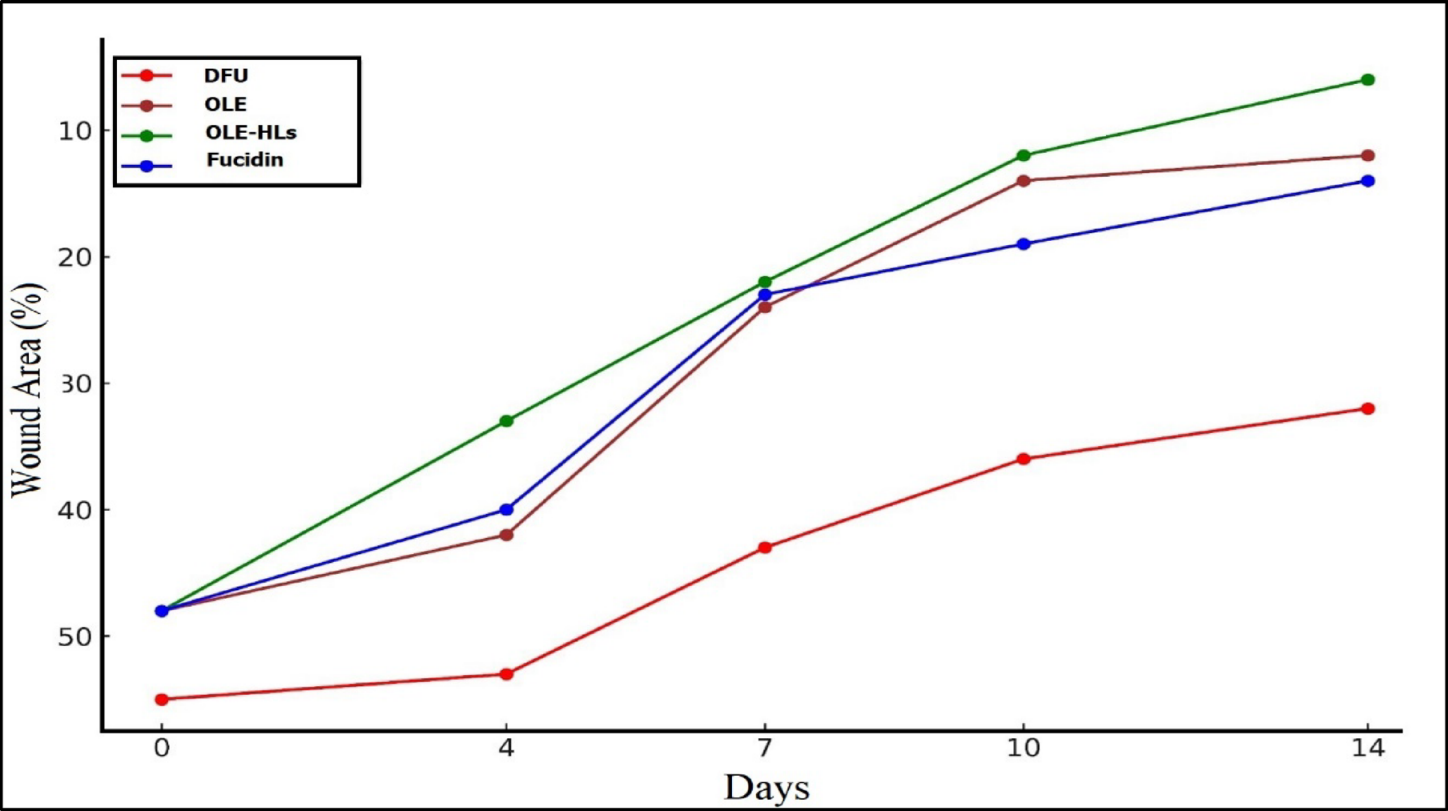
Wound closure progression in diabetic rats over 14 days under different treatments.

As shown in Figs. 8 and 9, the macroscopic evaluation of wound healing indicated a significantly enhanced repair response in diabetic rats treated with free OLE gel, OLE-HLs gel, and Fucidin cream when compared to untreated diabetic controls. Representative images in Fig. 9 demonstrate a progressive reduction in wound size across all treated groups, with OLE-HLs gel showing the most significant improvement, which exhibited substantial wound contraction by day 7 and nearly complete closure by day 14.

The superior healing observed with OLE-HLs gel is likely driven by the synergistic benefits of the nanocarrier system. The hyalurosome formulation enables sustained OLE release and improved bioavailability, while hyaluronic acid provides bioadhesive and hydrating properties. Together, these features create an optimal microenvironment for tissue regeneration, promoting reepithelialization, granulation tissue formation, fibroblast proliferation, and tissue remodeling. These findings align with earlier studies on oleuropein^69^ and nanocarrier-based wound therapies^74,75^. Positioning OLE-HLs gel demonstrates potential as a topical intervention for improving healing in diabetic chronic wounds.

### Biochemical and tissue markers

#### Metabolic profiling

As shown in Tables 4 and 5, the DFU group exhibited pronounced metabolic disturbances compared with the NC group. These included marked hyperglycemia, elevated glycated hemoglobin (HbA1c), and insulin resistance as indicated by higher HOMA-IR values, along with dyslipidemia characterized by dysregulated lipid profile. This pattern is consistent with previous reports in diabetic rat models, where a high-calorie diet (fat and carbohydrate) plus low-dose STZ induction resulted in similar persistent metabolic alterations^76^. Topical treatment with OLE gel, Fucidin, and OLE-HLs gel did not significantly improve glycemic parameters. Blood glucose and HbA1c remained elevated, insulin sensitivity was not restored, and persisted across all treated groups compared with NC. Meanwhile, only the OLE-HLs gel notably demonstrated a slight improvement in lipid profile. These findings indicate that, while OLE-based topical therapy may support local wound healing, it does not correct the systemic metabolic dysregulation underlying DFU. This reinforces the understanding that topical agents primarily act at the wound site and are unlikely to reverse systemic biochemical abnormalities without concurrent systemic interventions.

**Table 4:** Effect of OLE, OLE-HLs and Fucidin on Blood Glucose, Insulin, HOMA IR, and HBA1C in DFU treated rats.

Groups	Blood Glucose (mg/dL)	Insulin (ng/mL)	HOMA-IR (U)	HbA1c (%)
NC	92.4 ± 2.58ᵃ	3.1220 ± 0.2922ᵇ	0.70 ± 0.05ᵇ	3.96 ± 0.07ᵃ
DFU	225.4 ± 3.64ᵇ	0.2200 ± 0.0374ᵃ	0.11 ± 0.01ᵃ	6.14 ± 0.07ᵇ
OLE	230.2 ± 3.18ᵇ	0.3660 ± 0.0186ᵃ	0.20 ± 0.01ᵃ	6.08 ± 0.05ᵇ
OLE-HLs	230.8 ± 1.83ᵇ	0.3760 ± 0.0191ᵃ	0.21 ± 0.00ᵃ	6.06 ± 0.07ᵇ
Fucidin	229.4 ± 1.83ᵇ	0.3240 ± 0.0317ᵃ	0.18 ± 0.01ᵃ	6.26 ± 0.13ᵇ
*P* value	*P* < 0.05	*P* < 0.05	*P* < 0.05	*P* < 0.05

Data are expressed as mean ± SE (n = 5); means that share the same superscript symbol (s) are not significantly different, P < 0.05.

**Table 5:** Effect of OLE, OLE-HLs, and Fucidin on plasma cholesterol, triglycerides, and cholesterol-high-density lipoprotein (HDL) in DFU-treated rats.

Groups	Cholesterol (mg/dL)	Triglyceride (mg/dL)	HDL (mg/dL)	LDL direct (mg/dL)
NC Gp	110.8 ± 1.88ᵃ	92.8 ± 1.85ᵃ	36.0 ± 1.41ᵇ	55.68 ± 1.44ᵃ
DFU Gp	233.6 ± 2.14ᵇ	178.4 ± 3.19ᶜ	29.2 ± 0.86ᵃ	168.72 ± 3.06ᶜ
OLE Gp	230.4 ± 1.47ᵇ	172.4 ± 2.23ᶜ	28.4 ± 1.03ᵃ	167.32 ± 1.71ᶜ
OLE-HLs Gp	222.0 ± 2.61ᶜ	165.0 ± 0.89ᵇ	33.2 ± 1.02ᵇ	157.4 ± 3.86ᵇ
Fucidin Gp	222.4 ± 1.43ᶜ	170.4 ± 1.72ᶜ	29.0 ± 1.00ᵃ	162.32 ± 1.76ᶜ
*P* value	*P* < 0.05	*P* < 0.05	*P* < 0.05	*P* < 0.05

Data are expressed as mean ± SE (n = 5); means that share the same superscript symbol (s) are not significantly different, *P* < 0.05.

#### Modulatory effects of OLE-HLs on oxidative stress biomarkers

**Fig. 10:**
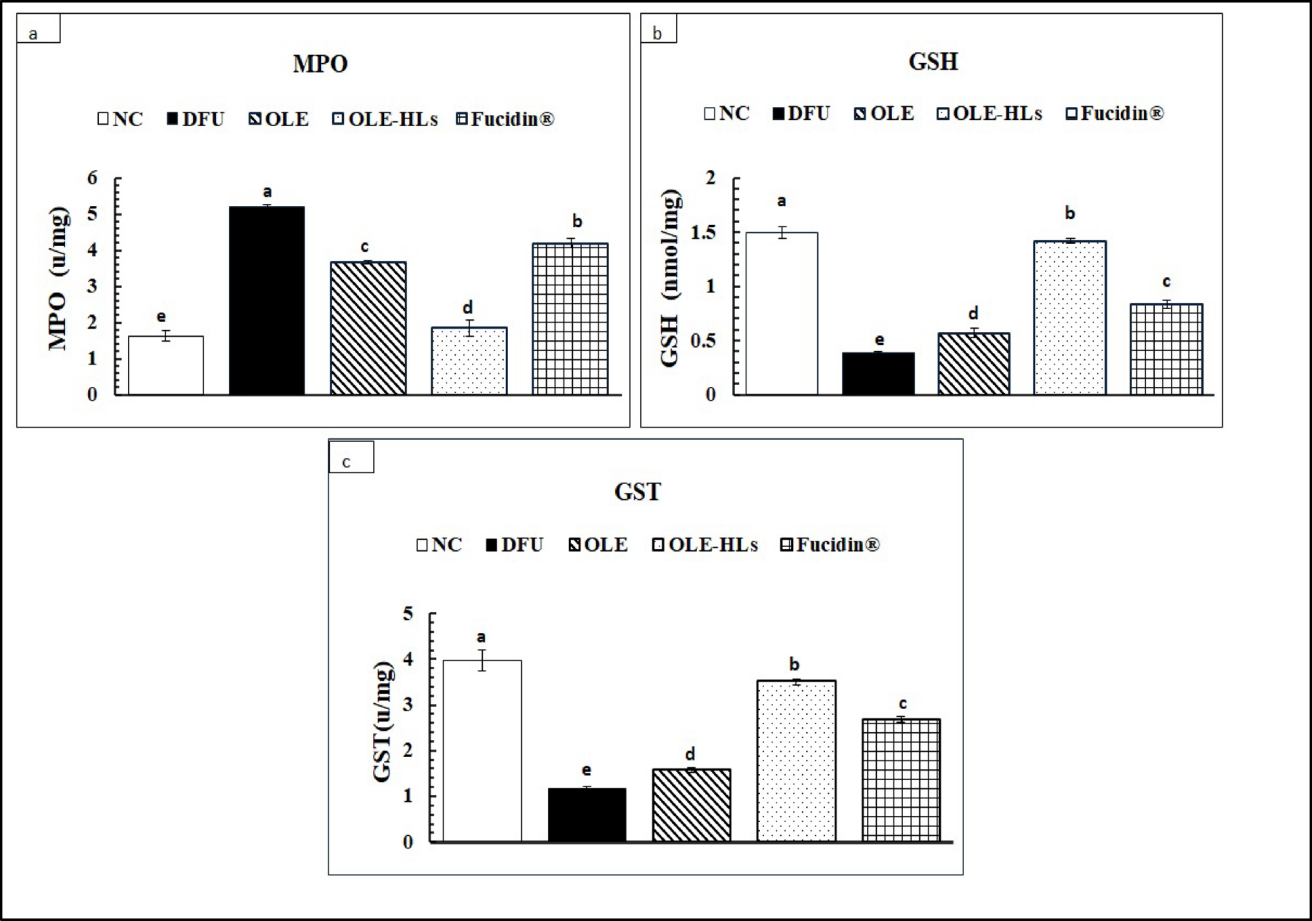
Effect of OLE, OLE-HLs and Fucidin on oxidative stress indicators: (**a**) MPO, (**b**) GSH, and (**c**) GST. Data are expressed as mean ± SE, means which share the same superscript symbol(s) are not significantly different, *P* < 0.05.

The DFU group exhibited notably decreased levels of GSH and GST, whereas MPO activity was markedly elevated relative to the NC group (*p* < 0.05; Fig. 10), reflecting severe oxidative stress and compromised antioxidant defenses. These alterations establish a hostile redox-inflammatory microenvironment that contributes to delayed wound healing, as shown in previous studies^77,78^. GSH depletion, reduced GST activity, and MPO overactivation exacerbate oxidative damage and inflammation, further impeding cellular repair and angiogenesis in chronic diabetic wounds^77,79^.

OLE-HLs gel exhibited the most significant improvement in redox balance compared to free OLE gel and Fucidin cream, owing to the hyalurosomal nanocarrier’s stability, enhanced tissue penetration, and sustained release, optimizing localized antioxidant action and supporting tissue repair^73^. The OLE-HLs gel formulation outperformed free OLE gel, which, despite effectively scavenging reactive oxygen species (ROS) and enhancing antioxidant defenses, showed minimal improvements due to poor solubility, instability, and low bioavailability. Combined with the intrinsic activity of oleuropein, which has been shown to replenish glutathione (GSH) by promoting its regeneration, increase glutathione S-transferase (GST) activity through Nrf2 activation, and reduce myeloperoxidase (MPO) activity by inhibiting NF-κB signaling, HLs effectively modulate redox balance and inflammatory responses in diabetic wound models^80–82^. Together, these effects enhanced localized antioxidant delivery, provided strong antioxidant protection, supported tissue repair, and highlighted the therapeutic potential of OLE-HLs gel in DFU and other oxidative stress–related chronic wounds.

These results reinforce the role of nanocarrier-based delivery systems in enhancing the pharmacological activity of natural bioactives like OLE and emphasize their clinical potential in diabetic wound management^83^. Furthermore, the suppression of MPO activity suggests a potential reduction in neutrophil-mediated inflammation, offering dual antioxidant and anti-inflammatory benefits.

#### Effect of OLE-HLs on inflammatory mediators and extracellular matrix-degrading enzymes:

**Fig. 11:**
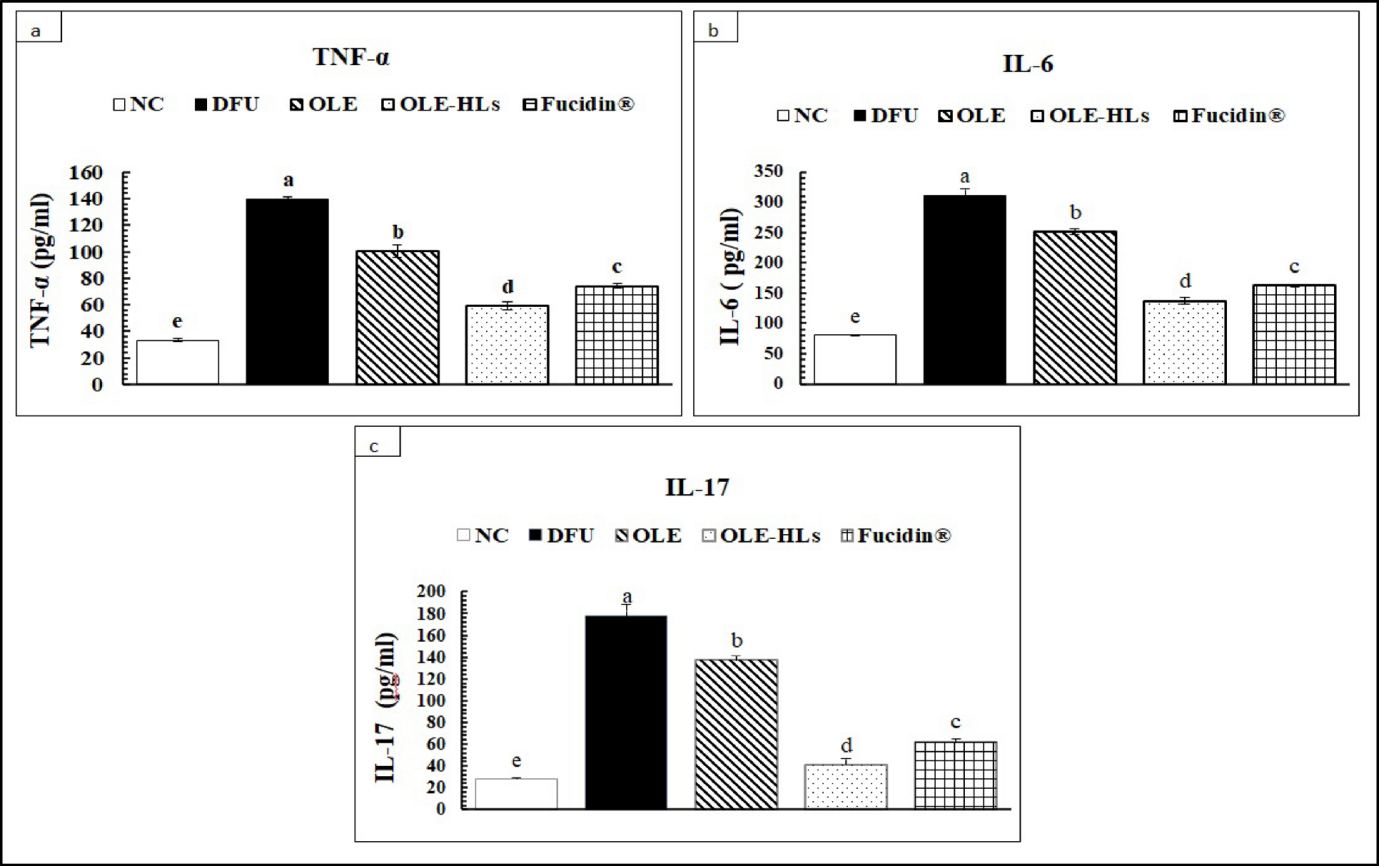
Effect of OLE, OLE-HLs and Fucidin on pro-inflammatory markers: (**a**) TNFα, (**b**) IL-6, and (**c**) IL-17. Data are expressed as mean ± SE, means which share the same superscript symbol(s) are not significantly different, *P* < 0.05.

**Fig. 12:**
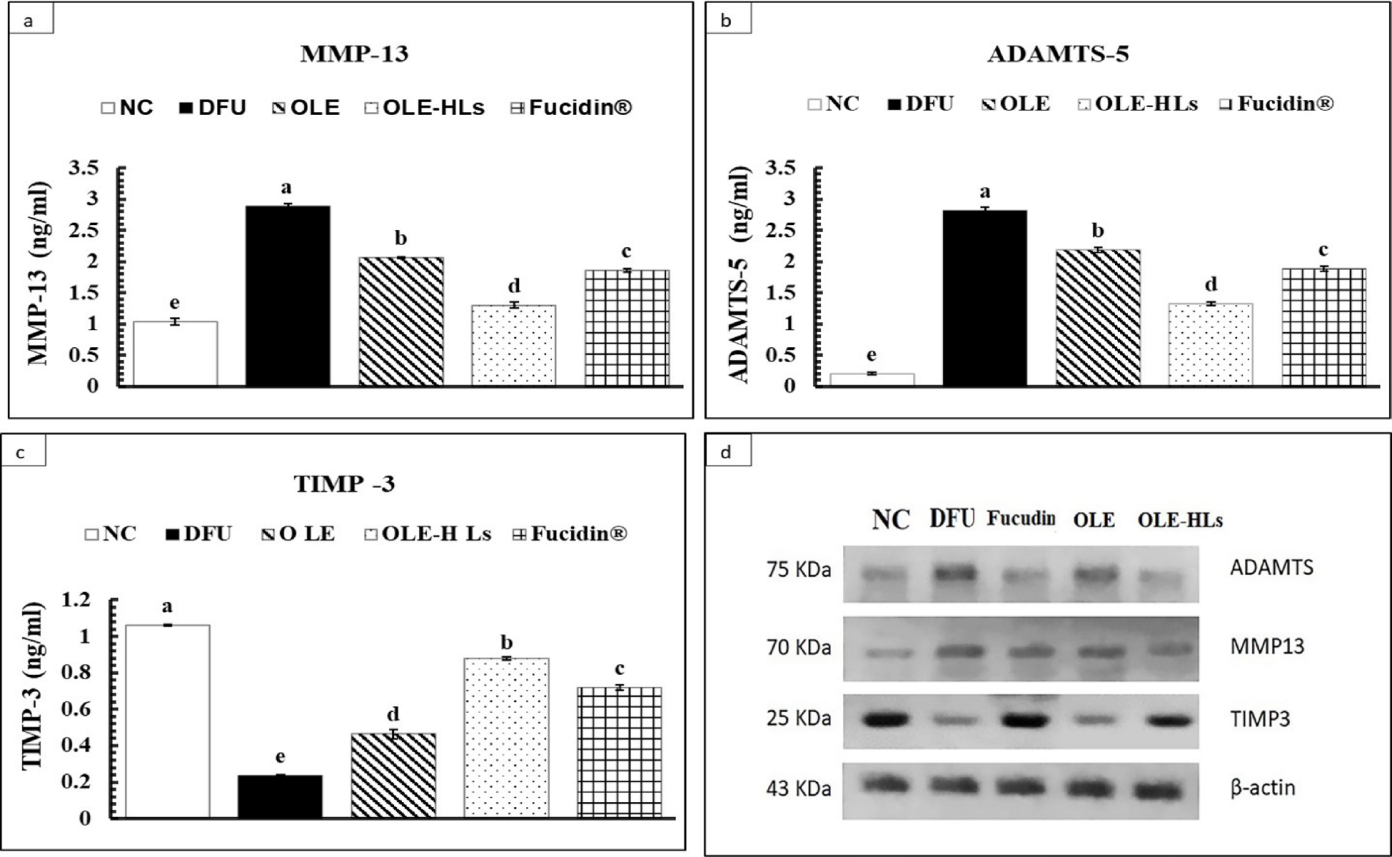
Effect of OLE, OLE-HLs, and Fucidin on extracellular enzymes: (**a**) MMP-13, (**b**) ADAMTS-5, and (**c**) TIMP-3, (**d**) western blot data. Data are expressed as mean ± SE; means which share the same superscript symbol(s) are not significantly different, *P* < 0.05.

As shown in Figs. 11 and 12, DFU tissues exhibited significantly elevated TNF-α, IL-6, and IL-17, with increased MMP-13 and ADAMTS-5 and reduced TIMP-3 expression (*p* < 0.05) compared to NC-GP, establishing a persistent cytokine–protease imbalance that promotes immune dysregulation, accelerates ECM degradation, impairs fibroblast and keratinocyte migration, suppresses angiogenesis, and ultimately perpetuates the non-healing state of DFUs^84^.

**Fig. 13:**
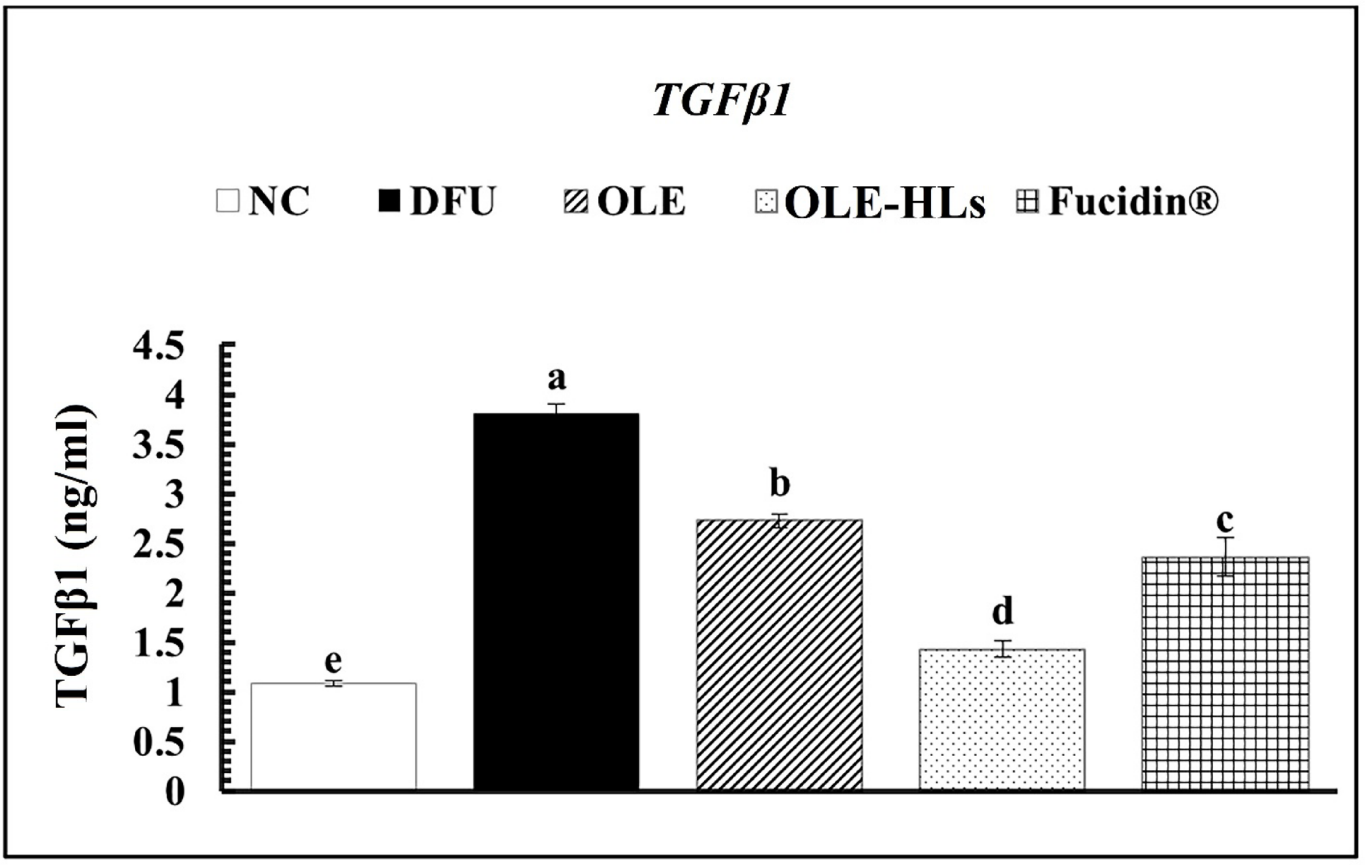
Effect of OLE, OLE-HLs and Fucidin on gene expression of *TGFβ1*in the skin tissue of rats. Data are expressed as mean ± SE, means which share the same superscript symbol(s) are not significantly different, *P* < 0.05.

Among the tested interventions, OLE-HLs gel exerted the most pronounced therapeutic effect, significantly downregulating cytokines and ECM-degrading enzymes while restoring TIMP-3 expression to near-control levels, thereby outperforming both OLE gel and Fucidin cream (Figs. 12, 13 and 1S). These results indicate that OLE-HLs simultaneously suppress inflammatory signaling and preserve ECM stability, facilitating the transition from a chronic to a regenerative wound state.

**Fig. 14:**
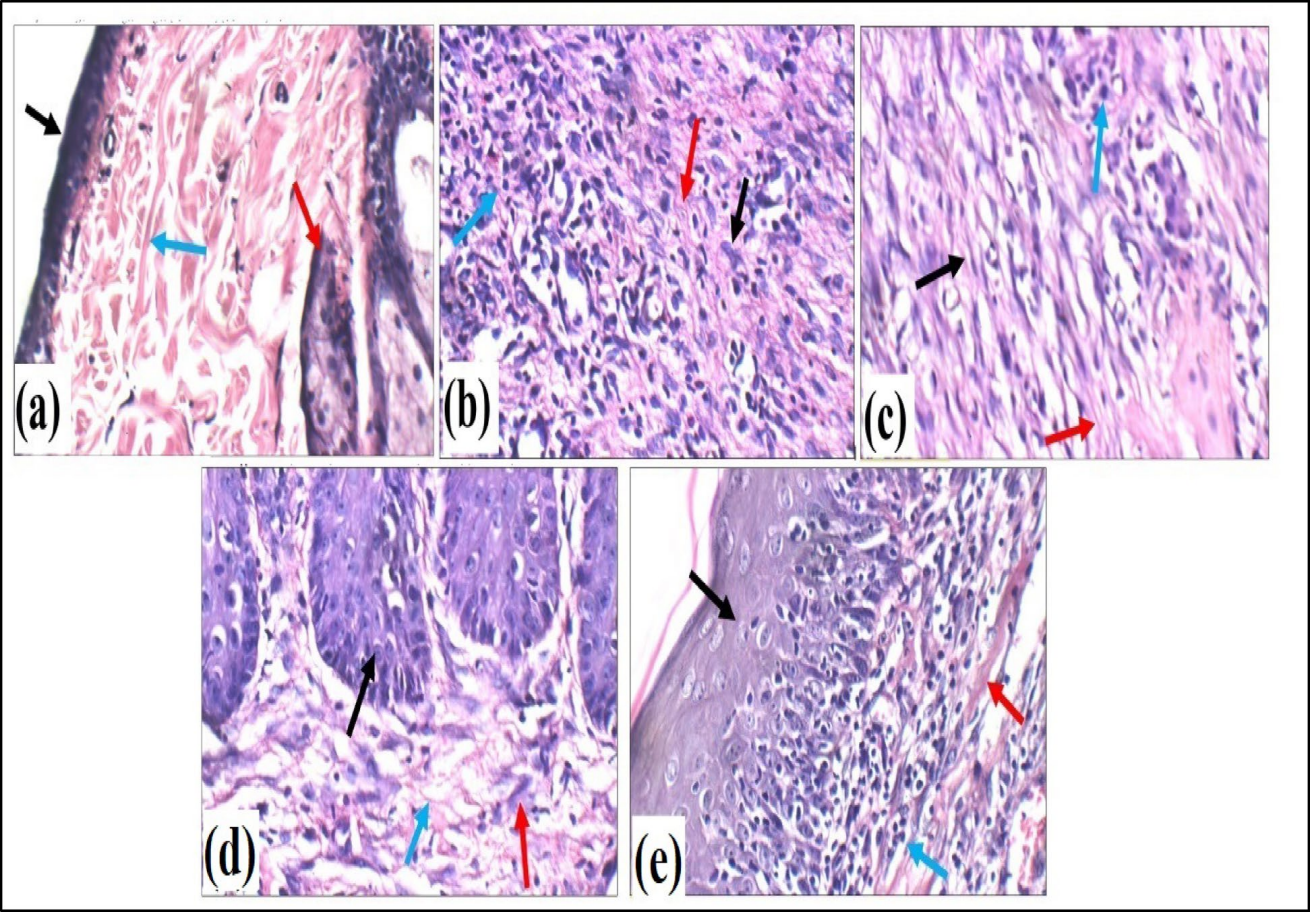
Histopathological Evaluation of Skin across Experimental Groups (H&E X400). (**a**) Normal control—GP: high power view showing average keratinized epidermis (black arrow), average pilosebaceous units (red arrow), and average collagen (blue arrow). (**b**) DFU—GP: high power view in deep dermis showing large scar tissue composed of excess active fibroblasts (black arrow), excess collagen (red arrow), and excess inflammatory infiltrate (blue arrow). (**c**) Fucidin cream—GP: high power view in upper dermis showing small scar tissue composed of inactive fibroblasts (black arrow), scanty thin collagen (red arrow), and scattered inflammatory infiltrate (blue arrow). (**d**) OLE-HLs gel—GP: high power view showing intact epidermis (black arrow), small hypocellular scar tissue composed of few inactive fibroblasts (red arrow), and few thin collagen (blue arrow). (**e**) OLE gel—GP: high power view showing intact epidermis (black arrow), small hypocellular scar tissue showing few thick collagen (red arrow), and few inflammatory infiltrate (blue arrow).

Mechanistically, the superior efficacy of OLE-HLs gel can be attributed to the combined effects of OLE and the nanocarrier system. OLE inhibits NF-κB signaling, thereby suppressing proinflammatory cytokines, which in turn prevents the upregulation of MMP-13 and ADAMTS-5 while preserving TIMP-3, leading to stabilization of ECM turnover and attenuation of chronic inflammation^74,85,86^. In parallel, the nanocarrier enhances OLE delivery through improved stability, sustained release, and bioavailability, while the hyaluronic acid backbone provides additional regenerative support by reducing neutrophil infiltration and stimulating fibroblast migration, angiogenesis, ECM remodeling, and granulation tissue formation^87^. Together, these combined effects attenuate inflammation, preserve ECM integrity, and explain the superior wound-healing outcomes of OLE-HLs.

Our findings demonstrate that OLE-HLs can both suppress chronic inflammation and preserve ECM integrity, which is consistent with previous reports on polyphenol-loaded hyaluronic acid hydrogels^88–90^. This dual action underscores their potential as a multifunctional nanoplatform capable of reprogramming the DFU microenvironment from a destructive to a regenerative state, providing a promising therapeutic avenue for chronic wound management.

**Fig. 15:**
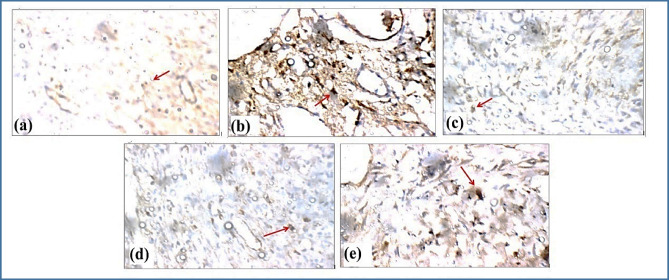
Immunohistochemical Expression of NF-κB in Skin Tissue (Immunostain, X400). (**a**) Normal Control-GP: Skin view showing negative reactivity (0) for NF-KB (red arrow). (**b**) DFU—GP: Skin view showing marked reactivity (+++) for NF-KB (red arrow). (**c**) Fucidin cream—GP: Skin view showing mild reactivity (+) for NF-KB (red arrow). (**d**) OLE-HLs gel—GP: Skin view showing mild reactivity (+) for NF-KB (red arrow). (**e**) OLE gel—GP: Skin view showing moderate reactivity (++) for NF-KB (red arrow).The criteria for assigning these scores were based on the intensity and distribution of the immunostaining observed under a microscope. Specifically:

#### Regulation of gene expression profiles by OLE-HLs

To investigate the molecular mechanisms underlying the therapeutic efficacy of the OLE-HLs nano-formulation, we assessed *TGFβ1* expression in diabetic wound tissues. In our study, *TGFβ1* expression was markedly elevated in DFU tissues compared with the NC group, confirming its role in sustaining the chronic wound environment (Fig. 13). Diabetic wounds are characterized by persistent inflammation and impaired tissue regeneration, closely linked to dysregulated *TGFβ1* signaling that alters immune responses, fibroblast activity, and extracellular matrix (ECM) remodeling^91^. Although *TGFβ1* is indispensable across all phases of wound repair, its persistent overexpression drives aberrant fibroblast activation, excessive collagen deposition, and delayed re-epithelialization, ultimately promoting fibrotic remodeling^92^.

Among treatment groups, OLE-HLs gel produced the most pronounced reduction in *TGFβ1* expression, restoring levels toward those of healthy skin and rebalancing a signaling axis implicated in chronic inflammation and fibrosis (Fig. 14). These effects are attributable to OLE-HLs’s antioxidant and anti-inflammatory features, reducing oxidative stress and fine-tuning growth factor signaling. By dampening excessive *TGFβ1*/Smad activity, OLE limits fibroblast overactivation and pathological collagen accumulation while preserving *TGFβ1*’s pro-healing functions. The enhanced efficacy of OLE-HLs over free OLE and Fucidin is likely due to the hyalurosomal carrier, which improves penetration, prolongs retention, and provides controlled release, thereby increasing local bioavailability. This improved delivery ensures effective modulation of *TGFβ1*, enabling balanced re-epithelialization and ECM remodeling without maladaptive scarring.

Our observations corroborate previous reports that Olea *europaea* extracts primarily attenuate pro-inflammatory gene expression via their major bioactive OLE, which enhances fibroblast proliferation, angiogenesis, and collagen deposition, and regulates key growth factors, including *TGFβ1*^93–96^. Our results indicate that OLE-HLs restore *TGFβ1*signaling to physiological levels and represent a promising therapeutic approach for chronic diabetic wounds. Finally, the OLE-HLs gel demonstrated exceptional stability and uniformity, indicating its potential for prolonged diabetic wound healing. Nanoencapsulation enhances colloidal stability; nonetheless, the long-term biocompatibility in individuals with diabetes necessitates additional research. Subsequent research should concentrate on enhancing bioavailability and release characteristics, investigating synergistic combinations with additional bioactive compounds, and conducting preclinical and clinical trials to assess safety, efficacy, and translational viability.

### Histopathological evaluation

As shown in Fig. 14 and Table 1s, histopathological analysis revealed that the DFU group exhibited severe pathological alterations, including epidermal atrophy, large hypercellular scar tissue devoid of adnexal structures, excessive fibroblast proliferation, thick collagen deposition, intense inflammatory infiltration, and proliferating small blood vessels, compared with the normal skin architecture of the control group, which showed intact keratinized epidermis, an organized dermis with pilosebaceous units, thin collagen bundles, abundant fibroblasts, normal vasculature, and underlying muscle. Treatment with fucidin cream or free OLE gel produced partial improvements, including restoration of epidermal thickness, improved collagen organization, reduced inflammatory infiltration, and evidence of vascular proliferation, although adnexal structures and full dermal integrity remained compromised. In contrast, OLE-HLs treatment achieved near-complete restoration of skin integrity, characterized by normal epidermal and dermal organization, dense and well-aligned collagen fibers, abundant fibroblasts, and minimal inflammatory infiltration, closely resembling the normal control group. These results are consistent with biochemical outcomes and previous reports showing that oleuropein promotes collagen deposition, fibroblast proliferation, angiogenesis, and suppression of inflammatory cell infiltration during wound healing^69^. The superior regenerative efficacy of OLE-HLs compared with free OLE is likely due to the nanocarrier’s enhanced stability, dermal penetration, and sustained release, which maximize local antioxidant and anti-inflammatory actions^97^. Moreover, the incorporation of hyaluronic acid provides synergistic benefits by attenuating oxidative stress, suppressing inflammatory mediators, stimulating fibroblast and keratinocyte migration, and modulating the wound microenvironment^98^. The combined actions of oleuropein and hyaluronic acid within the hyalurosomal matrix underpin the potent regenerative response observed in OLE–HLs–treated animals, highlighting this formulation as a promising therapeutic strategy for DFU and other chronic wounds.

### NF-κB immunohistochemical expression

NF-κB expression differed significantly among treatment groups, with the negative control showing no staining (score 0) and the positive control displaying strong nuclear and cytoplasmic reactivity (+++), confirming assay sensitivity. Both Fucidin cream and OLE-HLs gel treatments produced only mild positivity (+), indicating effective suppression of NF-κB activation, whereas OLE gel induced moderate expression (++), reflecting weaker regulation of inflammation (Fig. 15). These findings highlight the pathogenic role of NF-κB as a master regulator of pro-inflammatory cytokines (e.g., TNF-α, IL-6) and adhesion molecules that sustain leukocyte infiltration and tissue injury, ultimately delaying healing. By attenuating NF-κB signaling, OLE-HLs disrupt this inflammatory cascade, reduce oxidative stress, and foster a pro-resolving environment conducive to wound repair. The comparable efficacy of OLE-HLs to Fucidin underscores the therapeutic value of nano-encapsulation, which enhances bioavailability, dermal penetration, and sustained release, thereby offering a promising strategy for improving phytochemical-based interventions in inflammation and wound healing. These findings align with previous studies reporting that nano-encapsulation enhances bioavailability, enables targeted delivery, and reduces pro-inflammatory signaling, thereby offering potential benefits for skin repair^99,100^.

- Negative (0): No detectable staining (panel a, Normal Control-GP). Mild (+): Weak but visible staining, confined to a small number of cells (panels c and d, Fucidin cream–GP, OLE-HLs gel–GP). Moderate (++): Staining evident in a larger number of cells, though still somewhat sparse (panel e, OLE gel-GP). Marked (+++): Strong and widespread staining across the tissue section (panel b, DFU-GP).

## Conclusion

The present study indicated that oleuropein was successfully encapsulated within hyalurosomes in a stable and well-characterized nanocarrier system (size 254.64 nm, zeta potential − 25.12 ± 2.18 mV, entrapment efficiency 89.61 ± 1.82%), with TEM revealing spherical, uniformly sized, and well-dispersed vesicles. The formulation of a biphasic release profile improved cytocompatibility and enhanced cellular migration in the scratch assay. In vivo, OLE-HLs markedly accelerated diabetic wound healing by reducing oxidative stress and inflammation. Molecular, histopathological, and NF-κB immunohistochemical analyses corroborated these therapeutic effects*.* Overall, the OLE-HLs topical gel achieved remarkable healing outcomes in a diabetic foot ulcer model, highlighting its promise for future clinical evaluation in chronic wound treatment.

## Supplementary Information

Below is the link to the electronic supplementary material.

Supplementary Material 1

Supplementary Material 2

## Data Availability

The datasets used and/or analysed during the current study are available from the corresponding author on reasonable request.
