# Supplementary material for: In vitro and in vivo evaluation of oleuropein loaded hyalurosomes for diabetic foot ulcer healing
Source: Sci Rep. 2026 Mar 26;16:10480. doi: 10.1038/s41598-026-42804-5 (PMC13031767; doi:10.1038/s41598-026-42804-5)
Supplement: Supplementary file 1 — Supplementary Material 1 [file 41598_2026_42804_MOESM1_ESM.docx]

# Table 1s: Application of Scoring System on Experimental Groups:

| Figure | Re-epithelialization | Inflammatory Cell Density | Collagen Deposition | Granulation Tissue |
| --- | --- | --- | --- | --- |
| (a) Normal control - GP | (Score 2) | (Score 1) | (Score 2) | (Score 1) |
| (b) DFU - GP | (Score 1) | (Score 3) | (Score 3) | (Score 2) |
| (c) Fucidin® cream - GP | (Score 3) | (Score 1) | (Score 2) | (Score 2) |
| (d) OLE-HLs gel- GP | (Score 2) | (Score 1) | (Score 2) | (Score 1) |
| (e) OLE gel- GP | (Score 1) | (Score 1) | (Score 1) | (Score 2) |

The following scoring system was applied to assess key features of wound healing in the experimental groups, including re-epithelialization thickness, inflammatory cell density, collagen deposition, and granulation tissue formation. Each feature was evaluated based on the observed tissue characteristics, and a score from 0 to 3 was assigned to represent the severity of each aspect of healing.

1. **Re-epithelialization Thickness**: **Score 0**: No re-epithelialization observed.**Score 1**: Thin re-epithelialized layer. **Score 2**: Moderate thickness of re-epithelialization.**Score 3**: Thick re-epithelialized layer.
2. **Inflammatory Cell Density**: **Score 0**: No inflammatory cells present.**Score 1**: Low density of inflammatory cells (few scattered cells). **Score 2**: Moderate density (some clustering of cells). **Score 3**: High density (many clustered cells).
3. **Collagen Deposition**:**Score 0**: No collagen deposition.**Score 1**: Low collagen deposition (sparse or disorganized collagen). **Score 2**: Moderate collagen deposition (organized or moderately thick). **Score 3**: High collagen deposition (thick, dense collagen).
4. **Granulation Tissue Formation**: **Score 0**: No granulation tissue observed. **Score 1**: Minimal granulation tissue.**Score 2**: Moderate amount of granulation tissue.**Score 3**: Large or well-formed granulation tissue.
