# Supplementary material for: In vitro and in vivo evaluation of oleuropein loaded hyalurosomes for diabetic foot ulcer healing
Source: Sci Rep. 2026 Mar 26;16:10480. doi: 10.1038/s41598-026-42804-5 (PMC13031767; doi:10.1038/s41598-026-42804-5)
Supplement: Supplementary file 2 — Supplementary Material 2 [file 41598_2026_42804_MOESM2_ESM.docx]

Supplementary Fig. 1S(A-D): Uncropped full-length western blot membranes for Fig. 12d.”


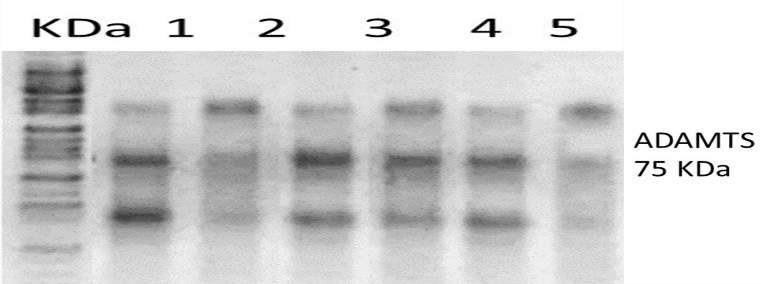


Fig. 1S(A): The uncropped data for ADAMTS are as follows: (1) Normal Control group (NC), (2) Diabetic Foot Ulcer group (DFU), (3) Fucidin group (Fucidin), (4) Oleuropein Gel group (OLE), and (5) Oleuropein-Hyalurosomes group (OLE-HLs).


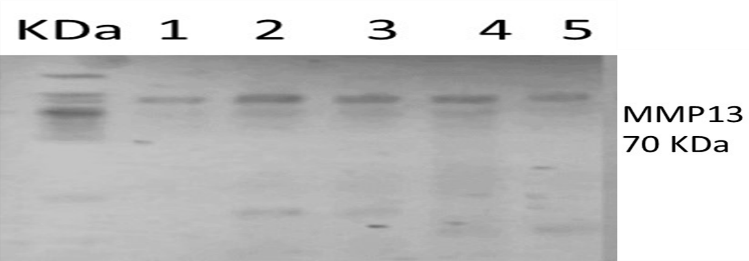


Fig. 1S(B): The uncropped data for MMP13 are as follows: (1) Normal Control group (NC), (2) Diabetic Foot Ulcer group (DFU), (3) Fucidin group (Fucidin), (4) Oleuropein Gel group (OLE), and (5) Oleuropein-Hyalurosomes group (OLE-HLs).


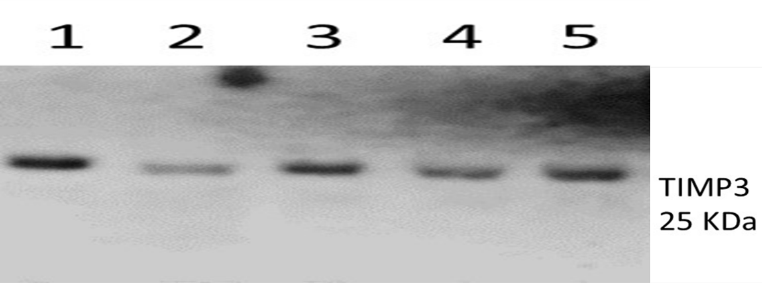


Fig. 1S(C): The uncropped data for TIMP3 are as follows: (1) Normal Control group (NC), (2) Diabetic Foot Ulcer group (DFU), (3) Fucidin group (Fucidin), (4) Oleuropein Gel group (OLE), and (5) Oleuropein-Hyalurosomes group (OLE-HLs).


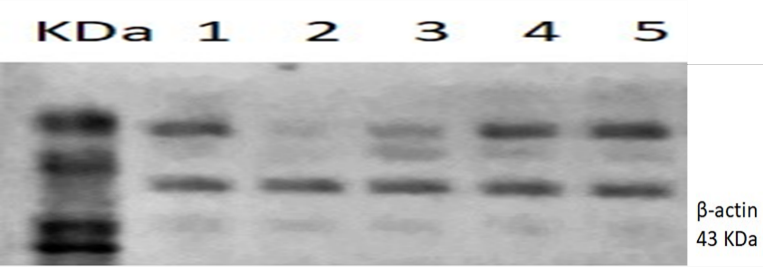


Fig. 1S(D): The uncropped data for β-actin are as follows: (1) Normal Control group (NC), (2) Diabetic Foot Ulcer group (DFU), (3) Fucidin group (Fucidin), (4) Oleuropein Gel group (OLE), and (5) Oleuropein-Hyalurosomes group (OLE-HLs).
